# A Peptide Filtering Relation Quantifies MHC Class I Peptide Optimization

**DOI:** 10.1371/journal.pcbi.1002144

**Published:** 2011-10-13

**Authors:** Neil Dalchau, Andrew Phillips, Leonard D. Goldstein, Mark Howarth, Luca Cardelli, Stephen Emmott, Tim Elliott, Joern M. Werner

**Affiliations:** 1 Biological Computation Group, Microsoft Research, Cambridge, United Kingdom; 2 Department of Applied Mathematics and Theoretical Physics, University of Cambridge, Centre for Mathematical Sciences, Cambridge, United Kingdom; 3 Faculty of Medicine, University of Southampton, Southampton, United Kingdom; 4 Department of Biochemistry, Oxford University, Oxford, United Kingdom; 5 Programming Principles and Tools Group, Microsoft Research, Cambridge, United Kingdom; 6 Institute for Life Sciences, University of Southampton, Southampton, United Kingdom; 7 School of Biological Sciences, Faculty of Natural Sciences and the Environment, University of Southampton, Southampton, United Kingdom; Massachusetts Institute of Technology, United States of America

## Abstract

Major Histocompatibility Complex (MHC) class I molecules enable cytotoxic T lymphocytes to destroy virus-infected or cancerous cells, thereby preventing disease progression. MHC class I molecules provide a snapshot of the contents of a cell by binding to protein fragments arising from intracellular protein turnover and presenting these fragments at the cell surface. Competing fragments (peptides) are selected for cell-surface presentation on the basis of their ability to form a stable complex with MHC class I, by a process known as *peptide optimization*. A better understanding of the optimization process is important for our understanding of immunodominance, the predominance of some T lymphocyte specificities over others, which can determine the efficacy of an immune response, the danger of immune evasion, and the success of vaccination strategies. In this paper we present a dynamical systems model of peptide optimization by MHC class I. We incorporate the chaperone molecule tapasin, which has been shown to enhance peptide optimization to different extents for different MHC class I alleles. Using a combination of published and novel experimental data to parameterize the model, we arrive at a relation of *peptide filtering*, which quantifies peptide optimization as a function of peptide supply and peptide unbinding rates. From this relation, we find that tapasin enhances peptide unbinding to improve peptide optimization without significantly delaying the transit of MHC to the cell surface, and differences in peptide optimization across MHC class I alleles can be explained by allele-specific differences in peptide binding. Importantly, our filtering relation may be used to dynamically predict the cell surface abundance of any number of competing peptides by MHC class I alleles, providing a quantitative basis to investigate viral infection or disease at the cellular level. We exemplify this by simulating optimization of the distribution of peptides derived from Human Immunodeficiency Virus Gag-Pol polyprotein.

## Author Summary

Major Histocompatibility Complex (MHC) class I molecules bind to protein fragments (peptides) within the cell and present these fragments at the cell surface, thus providing a snapshot of the cell contents that can subsequently be used to trigger an immune response. Only a fraction of the potentially billions of peptides inside a cell are selected for presentation, and the process is optimized to select for peptides that form a stable complex with MHC class I. The mechanisms of the optimization process are important for predicting the efficacy of an immune response and for designing effective vaccines, yet are still not well-understood. In this article we present a dynamical systems model of peptide optimization by MHC class I. We show that peptide optimization can be quantified and mechanistically explained by a peptide filtering relation, which relates cell surface abundance to peptide supply, peptide unbinding and interactions with the chaperone molecule tapasin. The filtering relation also accounts for differences in optimization across MHC class I alleles. Finally, we show how the filtering relation can be used to quantify the cell-surface presentation of virus-derived peptides for immune system surveillance.

## Introduction

**Figure 1:**
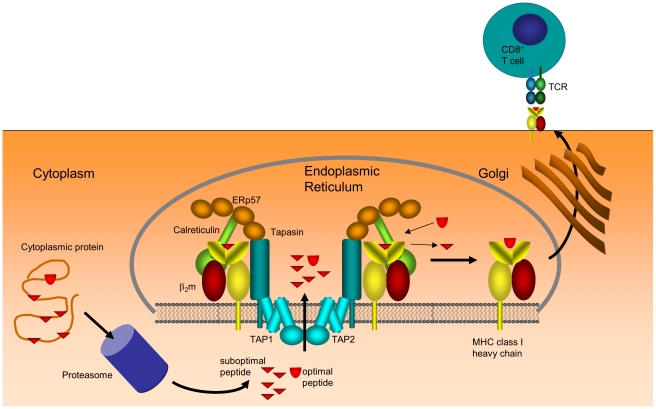
Basic process of MHC class I antigen presentation. Degradation of cytosolic and nuclear proteins, predominantly by the proteasome, generates peptides that are actively transported into the lumen of the endoplasmic reticulum (ER). Loading and editing of peptide cargo on MHC class I is achieved in the peptide loading complex, resulting in loaded MHC class I being released into the Golgi and transported to the cell surface, where the MHC class I peptide complex is presented to the immune system via the T-cell receptor. Known constituents of the peptide loading complex such as the transporter for antigen processing (TAP), tapasin, ERp57, calreticulin and MHC heavy-chain together with 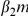 are shown explicitly.

MHC class I molecules are encoded within the genetic region known as the Major Histocompatibility Complex and are present in all nucleated human cells. MHC class I molecules direct cytotoxic T lymphocytes (CTL) to destroy virus-infected or cancerous cells, thereby preventing disease progression [1]. They provide a snapshot of the internal contents of a cell by binding to peptides arising from intracellular protein turnover and presenting these peptides at the cell surface, where the peptide-MHC complex can be recognized by CTL (Fig. 1). Most cells will present an array of tens of thousands of different peptides at their surface, some of which will be unique to virus-infected or cancerous cells. The efficacy of a CTL response to these peptides depends to a large extent on the ability of MHC class I molecules to select only a limited number of the potentially billions of different peptides that are generated by the hydrolysis of all intracellular proteins [2]. Peptides are selected for presentation on the basis of their ability to form a stable complex with MHC class I, by a process known as *peptide optimization*. A better understanding of the optimization of peptides is important for our understanding of *immunodominance* [1], the predominance of some CTL specificities over others, which can determine the efficacy of an immune response, the danger of immune evasion, and the success of vaccination and immunotherapeutic strategies.

Peptide binding to MHC class I occurs in the endoplasmic reticulum (ER) and is assisted by multiple cofactors that are thought to enable the optimization process [3]. The transporter associated with antigen processing (TAP) supplies the lumen of the ER with peptides generated in the cytosol and forms the backbone of the peptide loading complex (PLC). A number of chaperone molecules also comprise the PLC, namely calreticulin, calnexin, ERp57 and importantly tapasin, which bridges the gap between MHC class I and TAP [4] (Fig. 1). Of these chaperones, only tapasin is known to influence the extent of peptide optimization, in such a way as to skew the cell surface cargo towards peptides with low off-rates [5], and this influence is known to vary across different MHC class I alleles [6].

While a range of interactions between tapasin and MHC class I have previously been identified [7], [8], the effects of these interactions on peptide optimization are still not well-understood. A recent study used computational modeling to distinguish between different hypotheses of tapasin function within the ER [9], but the model assumed that only peptides with low off-rates could egress to the cell-surface, and was therefore unable to predict the optimization of peptides with different off-rates. As a result, the model was unable to account for observed effects of tapasin on peptide optimization, both over time [6] and at steady state [5].

In this paper we present a dynamical systems model for predicting MHC class I peptide optimization. We include interactions with the chaperone molecule tapasin, and propose a relation of *peptide filtering* to quantify peptide optimization as a function of peptide supply and peptide off-rates. Using a combination of published and novel experimental data, together with a combination of Bayesian inference and kinetic analysis, we show that tapasin can improve both the rate and extent of peptide optimization by accelerating peptide off-rate, and that differences in *optimization* across MHC class I alleles can be explained by an allele-specific peptide on-rate. Our filtering relation provides a mechanistic interpretation for recent experimental observations of peptide optimization both over time [6] and at steady state [5]. Finally, we demonstrate how the filtering relation can be used to quantify optimization of a large set of competing peptides in the context of an immune response, by simulating the cell surface abundance of Human Immunodeficiency Virus (HIV) peptides in complex with different MHC class I alleles.

## Results

### A model of MHC class I peptide optimization

**Figure 2:**
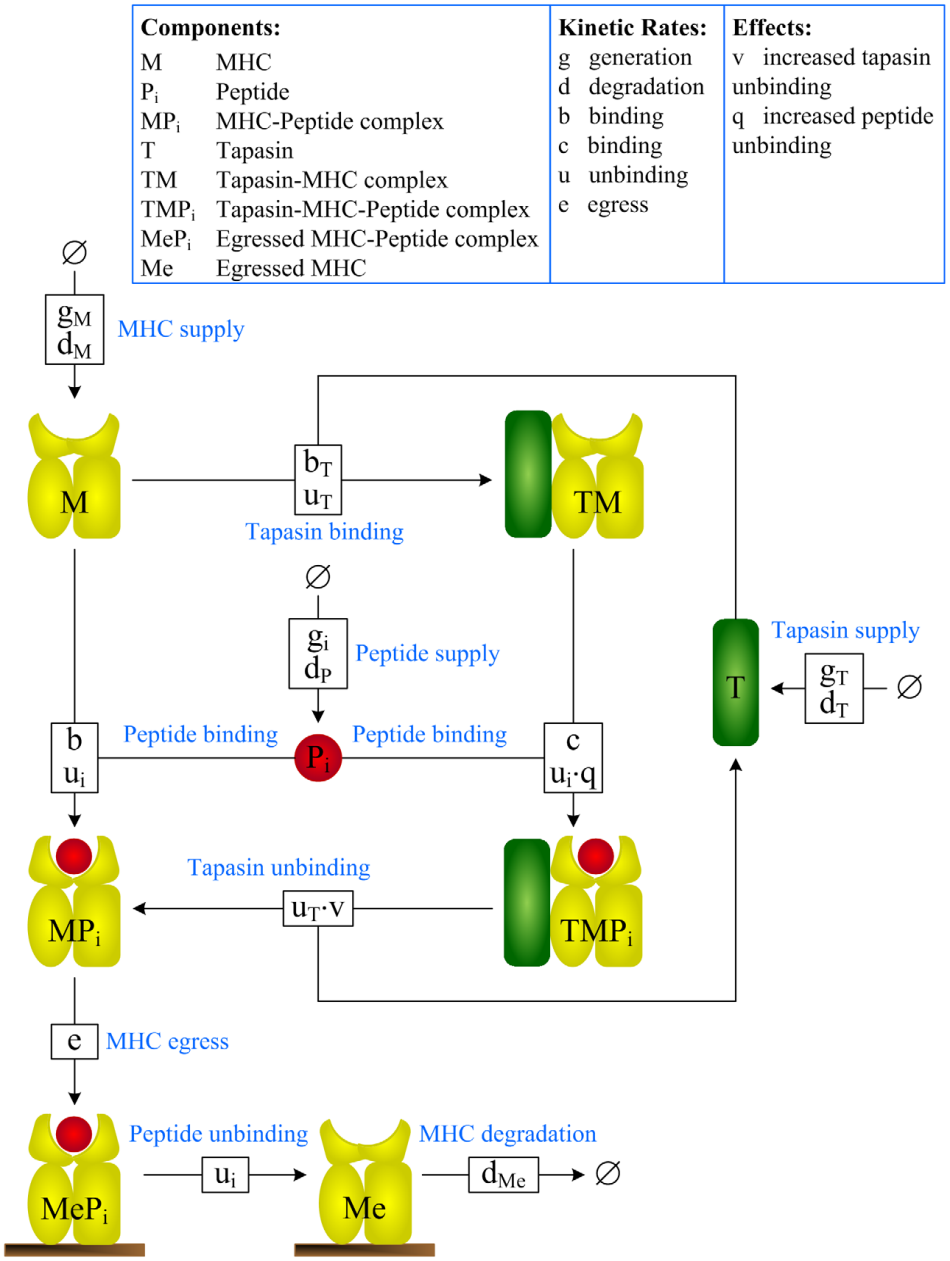
Indexed reactions for a dynamical systems model of MHC class I peptide optimization. Each shape in the model represents a molecular species and each box represents a reaction, where inbound edges represent reactants and outbound edges represent products. Boxes are labeled with corresponding reaction rates, where a single rate denotes an irreversible reaction and two rates denote a reversible reaction, with the rate of the forward reaction indicated on top. The subset of reactions taking place at the cell surface is given by 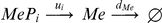 (see Methods for the full reaction set).

We formulated a dynamical systems model of MHC class I peptide optimization using a biological modeling language (SPiM [10], Fig. S1 in Text S1) and exported the model to an equivalent set of biochemical reactions for further analysis (Fig. 2). The model characterizes the interactions between MHC, peptides and tapasin within the endoplasmic reticulum, together with the dynamics of egressed peptide-MHC complexes at the cell surface.

A number of simplifying assumptions were made when constructing the model: (i) Peptides 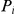 are supplied to the ER at rate 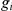 and then degraded or removed from the ER at rate 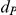. Since different peptides 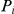 can have different levels of abundance within the cytoplasm and different rates of TAP transport, each peptide is associated with its own generation rate 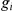. (ii) MHC class I heavy chain and 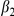
m are assumed to represent a single unit, where 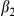
m dissociation from empty class I heavy chain is interpreted as a form of MHC degradation. (iii) All peptides are assumed to have a similar rate of binding 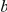 to MHC, such that *peptide affinity* is determined by a peptide-specific rate of unbinding 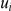, and is defined as 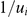. This is motivated by the measurements in [11]. (iv) Since MHC, tapasin and peptide continually cycle between the ER and Golgi apparatus [12], [13], we do not explicitly represent the Golgi as a separate compartment. Instead, we consider our ER compartment to include both the ER and Golgi, where the rate of egress 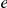 represents the rate of transit from the Golgi to the cell surface. By representing this process as a first-order reaction, we are making the simplifying assumption that the quantity of peptide-MHC complexes which egress is related to the quantity of complexes in the ER. (v) MHC can load peptides in the presence of tapasin at a higher rate 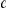, which implicitly models the stabilization of TAP molecules by tapasin, but we neglect egress of tapasin-bound MHC, since tapasin retains MHC by bridging it to the TAP transporter [14]. (vi) Tapasin can increase the rate of peptide unbinding from MHC by a factor 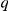 [8], while peptide can increase the rate of tapasin unbinding from MHC by a factor 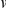 [15]. Tapasin has been shown to increase the peptide off-rate to a similar extent for peptides with a range of off-rates, though some variation has been shown for certain classes of peptide [8]. (vii) We neglect egress of empty MHC, which is retained and recycled in the ER by the chaperone calreticulin [16], [17]. (viii) Furthermore, we assume that 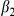
m dissociation from peptide-loaded or tapasin-bound class I heavy chain is negligible compared to 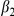
m dissociation from empty class I heavy chain. (ix) Once at the cell-surface, peptide unbinds from MHC irreversibly at rate 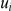, and empty MHC is degraded at rate 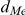. These assumptions can be refined in future iterations of the model.

### Predicting peptide optimization over time

MHC class I HLA-B alleles were previously shown to differ in their ability to optimize their peptide cargo over time, both in the presence and absence of tapasin [6]. Specifically, the HLA-B4402 (B4402) allele was shown to be highly dependent on tapasin for peptide optimization, while the HLA-B2705 (B2705) and HLA-B4405 (B4405) alleles were shown to be less tapasin-dependent. B2705 is of particular interest because it is a susceptibility factor for certain autoimmune diseases and is associated with long-term non-progression of HIV [18]. Therefore, we sought to use our peptide optimization model to explain the variation in tapasin-dependence between HLA-B alleles, through a combination of model simulation and Information Theory.

We simulated pulse-chase experiments [6] using the peptide optimization model of Fig. 2 , with representative peptides of low, medium and high affinity (Text S1). The experiments followed the thermostability of fixed cohorts of MHC class I complexes over time, making use of the known correlation between the thermostability of complexes and the affinity of their peptide cargo. Specifically, complexes stable at 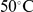 were shown to contain only high affinity peptides, complexes stable at 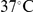 were shown to contain a combination of medium and high affinity peptides, while all complexes were shown to be stable at 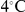, including empty MHC. Since the measurements correspond to both ER-localized and egressed peptide-MHC complexes, our assessment of the model was performed by comparing total peptide-MHC complexes with the 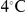 measurement, total medium and high affinity complexes with the 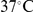 measurements, and total high affinity complexes with the 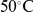 measurements. Since many of the kinetic parameters of the model have not previously been measured directly, due to the technical difficulties involved in obtaining such measurements, we used heuristic search methods to infer the parameter values from the experimental data [6] (see Methods). Essentially, this involved finding values for the parameters which minimized the deviation between the experimental data and the corresponding model simulation. Using this approach, we investigated how allelic variation in HLA-B might affect peptide optimization, by distinguishing between *allele parameters*, which were allowed to vary between alleles, and *fixed parameters*, which were assumed to be invariant between alleles. Each hypothesized set of allele parameters defined a variant of the model, which possessed a different intrinsic ability to reproduce the observed dynamics.

**Figure 3:**
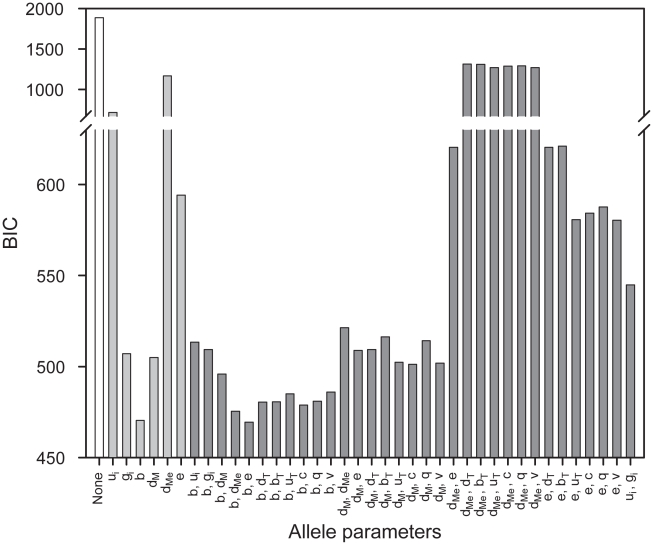
Selection of HLA–B allele parameters. The horizontal axis indicates the set of parameters that were allowed to vary between alleles. The vertical axis quantifies the Bayesian information criterion (BIC) of the best parameter values for a given set of allele parameters. BIC penalizes deviations of the model simulation from the experimental data, whilst also penalizing models with more variable parameters, implying that low BIC values correspond to more representative models. The best parameter values for a given set of allele parameters were inferred using the Filzbach MCMC software (see Methods).

**Figure 4:**
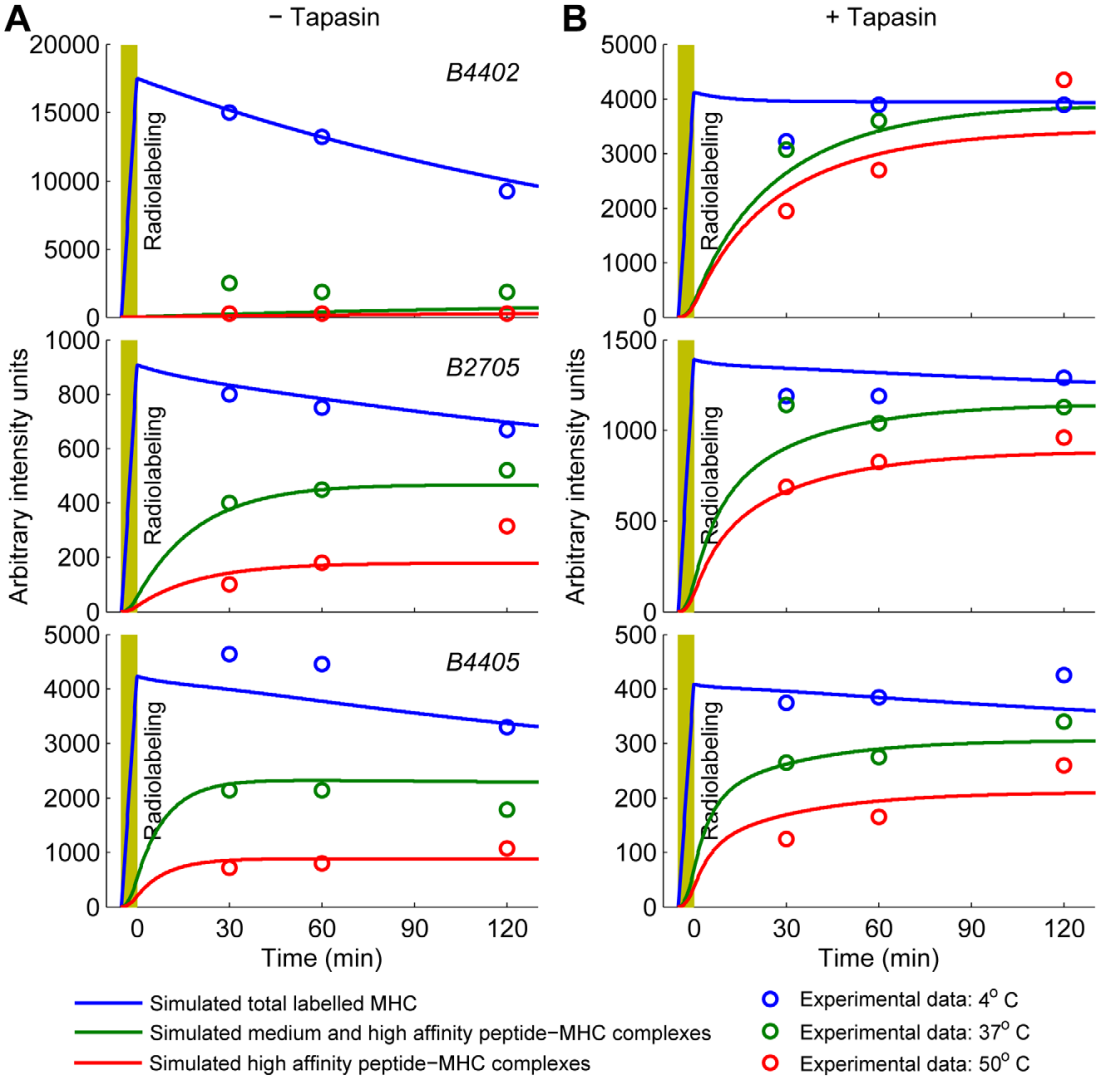
Simulation of time-dependent peptide optimization by HLA–B. The peptide optimization model of Fig. 2 was used to simulate a labeled cohort of peptide-MHC complexes by switching from generation of an unlabeled MHC population to a labeled population for 5 min (yellow blocks). The plots represent the concentration of total labeled MHC (blue), labeled MHC with medium or high affinity peptide (green) and labeled MHC with high affinity peptide only (red), at each time point. Simulations were performed in the absence (**A**) and presence (**B**) of tapasin. Corresponding experimental results [6] are also reported (circles). Simulations were conducted for representative low, medium and high affinity peptides with a separate dissociation rate 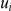 and generation rate 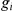 for each peptide 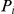, and a separate peptide binding rate 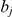 for each HLA–B allele 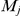 (Table S1 in Text S1; Protocol S1).

The Bayesian Information Criterion (BIC) [19] was used to quantify the performance of each set of allele parameters (equation (18) in Methods). BIC incorporates a term which penalizes the deviation of the simulation from the data, and a second term which penalizes increasing numbers of allele parameters. Therefore, BIC can be used to assess a range of models by taking into account the added cost of additional unconstrained variables. Since the dynamics of peptide optimization varied considerably between HLA–B alleles in the absence of tapasin [6], we reasoned that at least one allele parameter must be tapasin-independent. To incorporate this insight whilst focusing on the principal contributors to allelic variation, we examined combinations of up to two allele parameters, with at least one tapasin-independent parameter selected from 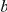, 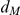, 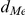, 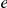, 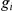 and 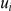.The best BIC scores were obtained when the peptide on-rate 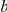 was the only allele parameter (470.38), and when both 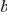 and the rate of egress 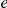 were the allele parameters (469.39; Fig. 3), suggesting that at least peptide on-rate is allele-specific. However, having both 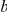 and 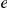 as allele parameters required unrealistically fast egress of B2705 and B4405 complexes to obtain a closer fit to the data (Fig. S2 in Text S1). Therefore a single allele parameter (Fig. 4) 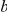 was used, which was able to effectively account for the experimental data [6]. Specifically, in the absence of tapasin B4402 exhibited worse time-dependent optimization than both B2705 and B4405 (Fig. 4 A), while in the presence of tapasin B4402 exhibited better time-dependent optimization than both of these alleles (Fig. 4 B). To ensure that the MCMC search algorithm was robust to random variations, and could reproducibly generate consistent parameter estimates, we produced 10 different chains for each model hypothesis. For the allele-specific 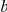 model, 8 out of 10 chains converged to BIC values between 470.38 and 470.69, while the other two chains performed poorly. We next plotted the mean and standard deviation of the posterior distributions of the model parameters for each of the 10 chains, which revealed that the 8 high performing chains had overlapping posterior distributions (Fig. S3 in Text S1), and were therefore producing consistent parameter estimates.

To understand the effects of an allele-specific peptide on-rate on peptide optimization, we plotted MHC complexes with high, medium, and low affinity peptide separately, and distinguished free and tapasin-bound MHC complexes within the ER and at the cell surface (Fig. S4 in Text S1). For B4402 without tapasin, an intrinsically low peptide on-rate meant that the majority of B4402 complexes remained in the ER without peptide, resulting in very low optimization. For B2705 and B4405 without tapasin, an intrinsically high peptide on-rate meant that these alleles rapidly bound their peptide cargo and exhibited good time-dependent optimization. In contrast, for B4402 with tapasin, most complexes first bound to tapasin and were subsequently able to rapidly bind peptides and optimize their peptide cargo, presenting almost exclusively high affinity peptides at the cell surface. For B2705 and B4405 with tapasin, the intrinsically high peptide on-rate meant that peptide out-competed tapasin for binding to MHC, such that a higher proportion of peptides followed the non-tapasin pathway, resulting in reduced optimization. Thus, variation in the intrinsic ability of free HLA–B alleles to bind peptide in the absence of tapasin was shown to be the most likely explanation for allelic variation in peptide optimization, both in the presence and absence of tapasin. For all three alleles, cell surface optimization could not be improved by modifying most other parameters in the model (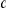, 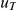, 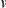, 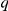 and 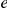) (Fig. S5 in Text S1). This indicates that the balance between peptide binding 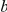 and tapasin binding 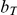 is a major determinant of peptide optimization, achieved by controlling the effectiveness of the tapasin-mediated pathway. The prediction that allele-specific tapasin dependency results from variations in peptide binding to MHC class I molecules is consistent with analysis from molecular dynamics simulations, which suggest that tapasin stabilizes peptide-receptive conformations [20]. This stabilization in the presence of tapasin is represented in our model by setting the binding rate 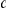 to be allele-independent and greater than or equal to the binding rate 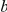. In the absence of tapasin, MHC class I molecules of different alleles may have varying levels of peptide receptiveness, which is represented in our model by allowing 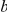 to vary between alleles.

### Kinetic control of peptide optimization

Having established a hypothesis which explains how MHC alleles experience differential tapasin-dependence, we sought to identify the mechanisms that determine the extent and rate of peptide optimization, both in the presence and absence of tapasin. Peptide optimization is the process by which high affinity peptides are selected for presentation at the cell surface [6]. Peptide-MHC complexes generally need to be stable for many hours or days at the cell surface in order to effectively elicit an immune response [21], yet peptide optimization in the ER is typically limited to tens of minutes [3], [6], [22]. This requires optimization beyond the limit that would be obtained in equilibrium. How such high optimization is achieved in so little time is still not well-understood [3].

One way to increase the extent of peptide optimization is for peptide-MHC complexes to be retained in the ER for an extended period prior to egress, so that unstable peptides have an opportunity to unbind [22]. However, delaying egress also increases the time for complexes to reach the cell surface. Therefore, a trade-off exists between the extent of optimization and the rate at which this optimization can be achieved. We quantify this trade-off by calculating the relative probabilities of MHC egress and peptide unbinding.

**Figure 5:**
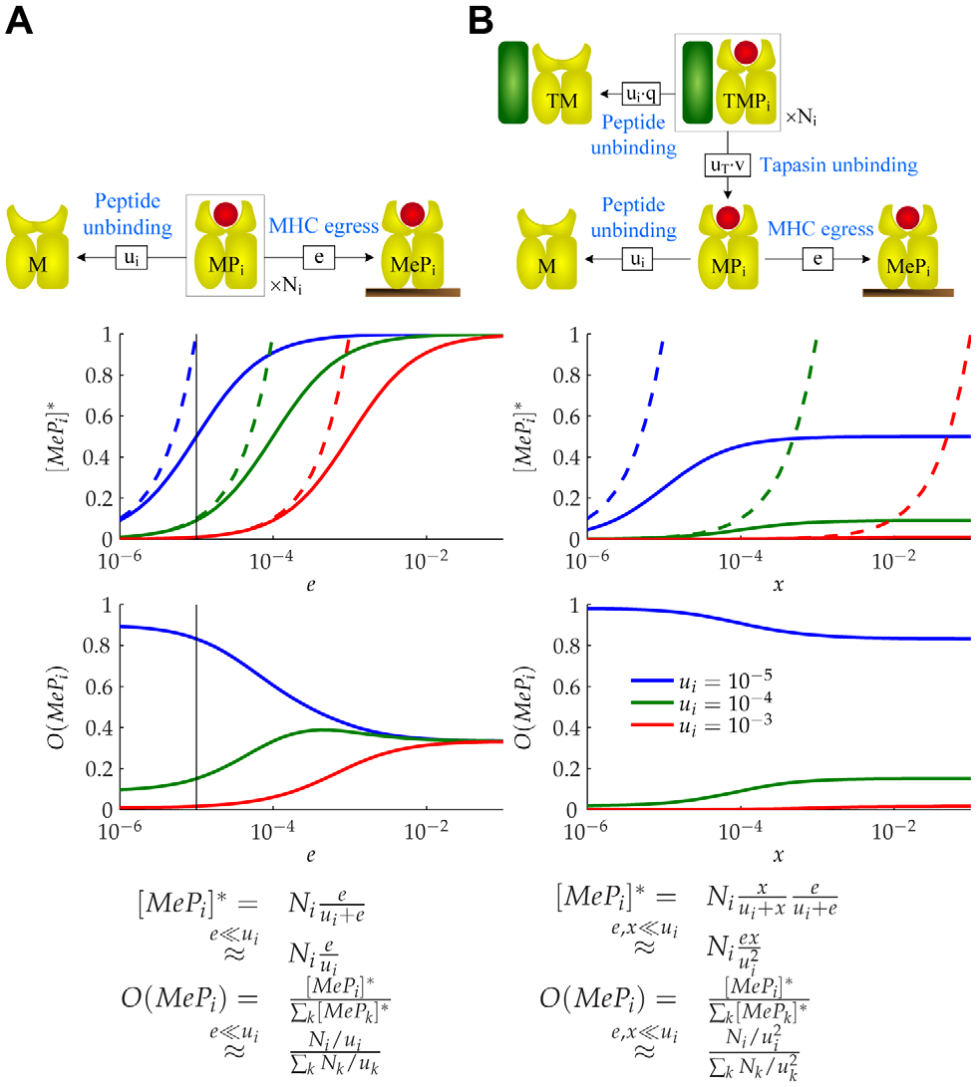
Filtering relation of MHC class I peptide optimization. (**A**) Consider a population of 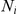 MHC complexes containing peptides with off-rate 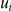. 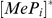 denotes the expected number of MHC complexes that will egress before the peptide can escape. 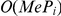 denotes the expected proportion of egressed MHC complexes that will contain peptides with off-rate 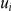. This defines a measure of peptide optimization. We plot 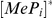 and 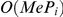 as functions of 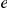 for three peptides with different off-rates and the same initial populations. Maximal optimization is achieved when 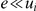, with 
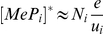
 (dashed lines). (**B**) Consider a population of 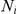 tapasin-MHC complexes containing peptides with off-rate 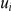. 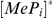 denotes the expected number of MHC complexes that will unbind from tapasin *and* egress before the peptide can escape, where 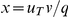. 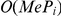 is defined as in A. We plot 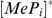 and 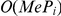 as functions of 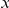 with 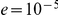 (black line in A). Maximal optimization is achieved when 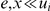, with 
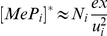
.

Consider an MHC complex containing a peptide with off-rate 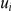 (Fig. 5 A). The complex can either egress to the cell surface at rate 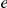, or the peptide can unbind at rate 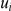. The probability of each event is proportional to its rate, such that the probability of egress is given by 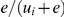. The competition between unbinding 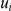 and egress 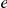 defines a *peptide filtering* step, where the basic filtering mechanism is comparable to principles of kinetic proof-reading [23]. Let 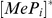 denote the expected number of peptide-MHC complexes that egress to the cell surface before the peptide can escape (Fig. 5 A). If there are 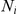 MHC complexes containing peptides with off-rate 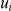 in the ER, we expect 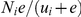 to egress and the remainder to release their peptide cargo. For very high 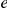, all 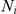 complexes will egress irrespective of their peptide cargo. For very low 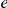, the number of egressed complexes will tend to 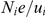. Let 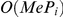 denote the proportion of egressed MHC complexes containing peptides with off-rate 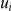 (Fig. 5 A). This defines a measure of *peptide optimization*. For very high egress we observe no optimization, where the proportion of peptides at the cell surface is equal to the proportion of peptides in the ER. For very low egress we observe maximum optimization, where the proportion of peptides at the cell surface varies inversely with the peptide off-rate.

The introduction of tapasin provides an additional filtering step (Fig. 5 B), involving a competition between peptide unbinding 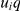 and tapasin unbinding 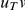. Let 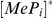 denote the expected number of MHC complexes that unbind from tapasin *and* egress to the cell surface before the peptide can escape (Fig. 5 B). For very high 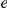 and 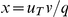, all 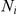 complexes will egress irrespective of their peptide cargo. For very low 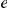 and 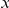, the number of egressed peptide-MHC complexes will tend to 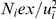. The number of egressed complexes therefore varies with 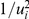 in the presence of tapasin (Fig. 5 B), compared with 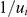 in the absence of tapasin (Fig. 5 A). This implies that tapasin enhances presentation according to peptide affinity 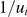, in agreement with experimental results [5]. Since the proportion of egressed complexes now varies inversely with the *square* of peptide off-rate, low affinity peptides are much more likely to escape than high affinity peptides, resulting in improved peptide optimization.

The peptide filtering relation presented above also holds for the full dynamical systems model of Fig. 2 , in which peptides can bind and unbind multiple times to MHC. By translating the reactions of Fig. 2 to a set of ordinary differential equations, we obtained the following expression for the steady-state concentration of peptide-MHC complexes at the cell surface (see Methods):
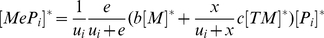
(1)


where 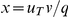. The equation includes the ER peptide filtering steps described in Fig. 5, together with peptide optimization at the cell surface given by 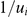, where peptides with a lower off-rate 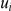 are more likely to remain bound to MHC. The equation also quantifies the ratio of egressed peptide-MHC complexes that are loaded in the presence and absence of tapasin, given by the ratio of 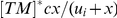 to 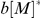. Assuming peptide loading takes place via the tapasin pathway (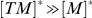) and that peptides have a high turnover in the ER [24], characterized by high generation and degradation rates (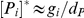), we can simplify equation (1) as
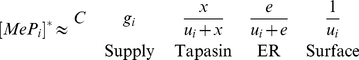
(2)


where 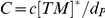. This corresponds to an upper bound on peptide optimization in the presence of tapasin. In the absence of tapasin, the equation for 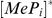 is the same as (2) but without the tapasin optimization step 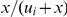. This implies that tapasin enhances peptide presentation according to peptide affinity 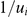, in agreement with the analysis of Fig. 5 and experimental findings [5].

To further place our insights in a biological context, we used the dynamical systems model to identify the mechanisms that determine the rate of peptide optimization. Consider the filtering step between peptide unbinding 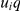 and tapasin unbinding 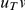. Tapasin can enhance peptide optimization to the same extent either by increasing the peptide off-rate by a given factor 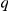, or by decreasing the tapasin unbinding rate by the same factor. However, decreasing the tapasin unbinding rate essentially delays the transit of MHC to the cell surface, resulting in slower optimization. In contrast, increasing the peptide off-rate allows tapasin to increase the extent of peptide optimization while still maintaining a rapid flux of peptide-MHC complexes to the cell surface.

### Predicting peptide optimization at steady-state

**Figure 6:**
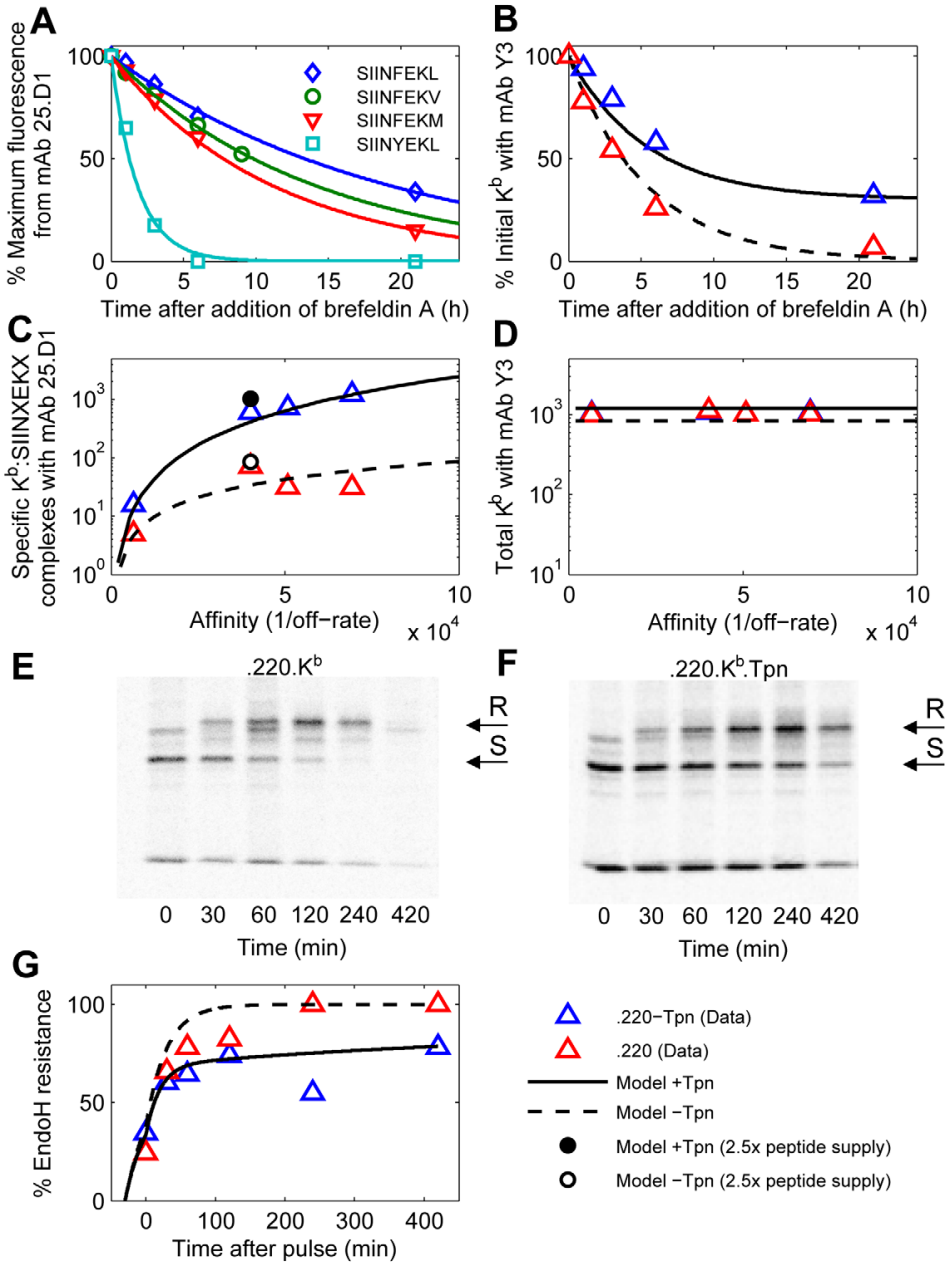
Peptide optimization and trafficking for H2−K. 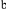
 The model of Fig. 2 was calibrated for H2−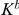 by varying the rates of peptide binding, MHC degradation at the cell surface, and egress (Table S2 in Text S1; Protocol S2). Each simulation computes the steady state of the model with three types of peptide: two background peptides 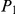 and 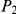 and one of the four SIINFEKL peptide variants 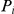 (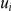 estimated from data in panel A). (**A**) Release of peptides from MHC following treatment with brefeldin A (BFA) measured with 25.D1 (symbols), fitted to single exponential decays (solid lines). (**B**) Dissociation of endogenous peptides from cells treated with BFA. (**C**) Steady-state presentation of specific peptide-MHC complexes at the cell surface, comparing simulation with measurements of 25.D1 from [5]. (**D**) Total steady-state peptide-MHC complexes (cell surface), comparing simulation with measurements of Y3 from [5]. Simulated values were scaled by a proportionality factor for optimally overlapping the 25.D1 data (with SIINFEKM removed) and the Y3 data (all points) (Text S1). (B–D) The x-axis shows the relative affinity of peptides given by the inverse of the off-rate. Steady state concentrations were obtained by equating the right hand sides of the ODEs to zero. Steady state concentrations in tapasin-deficient cells were simulated by setting 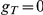. (**E–G**) For quantifying egression of peptide-MHC complexes, .220.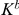 (E) and .220.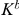.Tpn (F) were pulsed for 10 min with 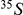-Met/Cys and chased for the indicated times (min). Y3 immunoprecipitates were digested with endoglycosidase-H (EndoH) and SDS-PAGE and autoradiography were performed. Arrows indicate 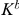 heavy chain resistant (R) and sensitive (S) to EndoH digestion. EndoH analysis of H2−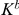 was performed as described previously [6]. (B–D, G) The solid lines indicate model simulations and triangles indicate measured data-points. The experimental data for (A,B,E–F) is novel, while the experimental data for (C,D) is from [5].

To further probe the applicability of our model, we investigated whether it could be used to predict peptide optimization at steady state. Previously, the effects of tapasin on steady-state peptide optimization were measured for peptides in the MHC class I allele H2−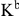 (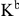) [5]. The experiments were conducted using four target peptides, obtained by performing substitutions at positions 5 and 8 of the amino acid sequence SIINFEKL. Peptide off-rates were measured in RMA-S cells and each of the target peptides were introduced as minigenes into a tapasin-deficient cell line (.220) and into the same cell line transfected with tapasin (.220.Tpn). Steady-state levels of cell surface peptide-MHC complexes were measured by flow cytometry using mAb 25.D1 (Fig. 6 C, Table S3 in Text S1), which specifically recognizes the SIINFEKL peptide variants bound to 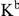 [25]. Total cell-surface MHC was also measured with mAb Y3, which recognizes empty and peptide-occupied 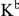 (Fig. 6 D, Table S3 in Text S1).

We complemented previous experimentation [5] by measuring the off-rates of the target peptides in .220 cells directly. Cells were treated with Brefeldin A (BFA) to prevent further MHC egress, allowing direct characterization of the dissociation of cell-surface MHC complexes carrying SIINFEKL peptide variants with 25.D1. The off-rates of the target peptides were estimated by fitting single exponential decays (SIINFEKL: 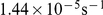, SIINFEKV: 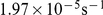, SIINFEKM: 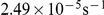, SIINYEKL: 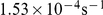; Fig. 6 A). We also used Y3 to measure total MHC during BFA incubation of cells with no target peptide, in the presence and absence of tapasin, to provide an indication of the off-rates of endogenous peptides presented by .220 and .220.Tpn cells (Fig. 6 B).

We simulated the above experiments using the peptide optimization model of Fig. 2 with a parameter set specific to H2−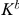 (Table S2 and Fig. S6 in Text S1). The full range of endogenous peptides was characterized by two representative peptides with off-rates 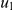 and 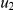, and supply rates 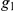 and 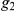 (Text S1). Each experiment was simulated using one of the target peptides with off-rate 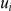, together with the representative endogenous peptides. Since target peptides were expressed at approximately equal levels inside cells [5], we assumed that they were generated at the same rate 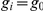. The model simulations agreed with the trends observed experimentally, accurately recapitulating the enhancement of steady state optimization conferred by tapasin (Fig. 6 C, D, Table S3 in Text S1). However, we observed that the model did not fit the experimental data for SIINFEKM as well as for the other target peptides. We hypothesized that the poor fit could be caused by increased TAP transport of the SIINFEKM peptide, due to a change in the terminal residue at position 8 [26]. To explore this idea, we increased the generation rate of SIINFEKM by a factor of 2.5, which gave a better fit to the experimental results (Fig. 6 D). This hypothesis further highlights the potential importance of peptide supply in predicting relative presentation levels [27], as can be seen from the peptide filtering relation (2). Although experimental measurements were only obtained for four distinct peptides, the model predicts the presentation levels for a continuum of peptide off-rates over a broad range, which can be checked in future experiments.

To distinguish between optimization resulting from delayed tapasin unbinding versus enhanced peptide off-rate, we measured the time taken for a fixed cohort of pulse-labeled MHC complexes to reach the cell surface by measuring endoglycosidase-H (EndoH) resistance (Fig. 6 E–G). By taking into account the temporal constraints of the EndoH data, we found that enhanced peptide off-rates were required to allow increased peptide optimization in the presence of tapasin without significantly delaying the transit of peptide-MHC complexes to the cell surface (see Fig. S7 in Text S1). Further parameter variation analysis indicated that cell surface optimization is nearly maximal in the H2−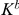 model with respect to 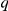, 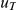, 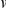 and 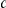, but could be improved by reducing 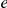 (Fig. S8 in Text S1). However, reducing 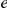 decreases the export of peptide-MHC complexes, suggesting a possible trade-off between optimization and the efflux of new information concerning cellular protein content.

### Predicting optimization of viral peptides

**Figure 7:**
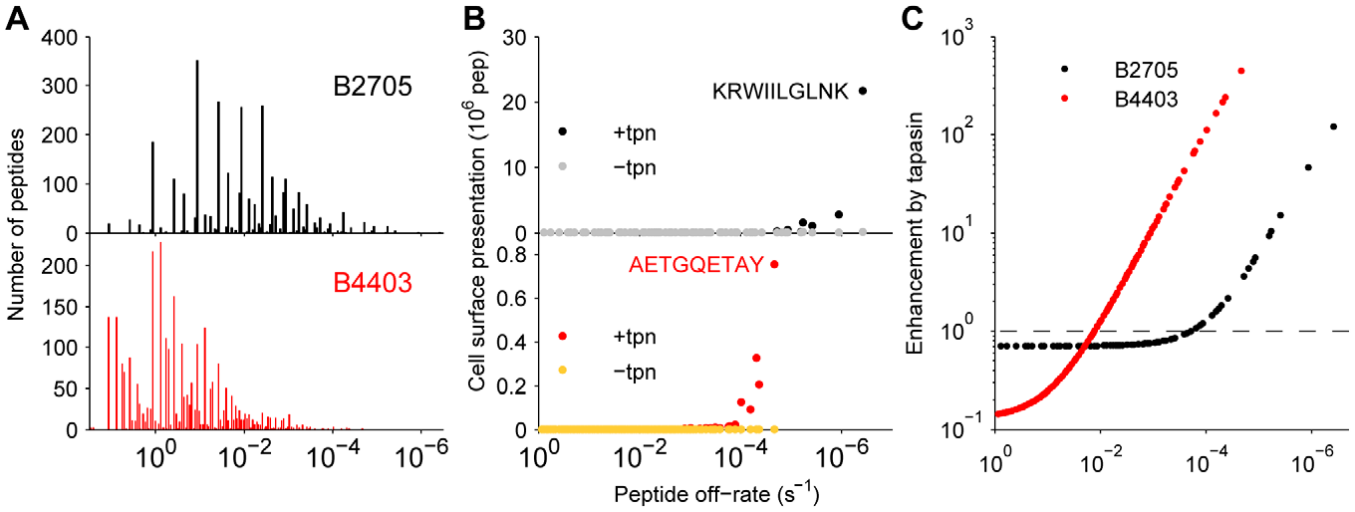
Simulation of cell surface presentation of HIV virus peptides by HLA–B2705. The sequence of the HIV-1 polyprotein Gag-Pol was obtained from the UniProt online resource (accession P03367). All peptides between 8 and 10 amino acids in length were then derived from the sequence and assessed for their off-rates using the BIMAS prediction algorithm (http://www-bimas.cit.nih.gov/molbio/hla_bind [28]). The peptides were then simulated by assuming that they are all supplied into the ER via TAP at an equal rate, such that the total supply rate is equal to the total supply rate estimated for Fig. 4. As the algorithm predicted many peptides to have the same off-rate, peptides were clustered for ease of computation. (**A**) The number of peptides with a given peptide off-rate, as calculated by BIMAS. (**B**) Steady-state cell surface presentation of peptide-MHC complexes as a function of peptide off-rate. Peptide supply was assumed to be constant for each individual peptide. Therefore, the supply rate associated with a particular off-rate is simply scaled by the number of peptides with that off-rate, as quantified in A. The lowest off-rate (highest affinity) peptides for B2705 (KRWIILGLNK) and B4403 (AETQCETAY) are indicated. Simulations were performed for the presence and absence of tapasin, as indicated in the key. (**C**) Enhancement of cell surface presentation by tapasin was computed by dividing simulated tapasin-sufficient presentation by simulated tapasin-deficient presentation for each peptide. The results of the HIV simulations illustrate the extent to which tapasin can affect a downstream immune response. Theoretically, tapasin can enhance presentation by up to a factor 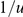, where 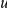 is the off-rate of the peptide from MHC (Fig. 5). However, the characteristics of the MHC allele, such as the allele-specific peptide on-rate, can significantly alter the effect of tapasin on the presentation of a given peptide. Our model allows differences in presentation levels to be quantified by taking into account peptide supply and peptide off-rate, together with the effects of tapasin and the binding properties of the MHC class I allele under consideration. In particular, our analysis of the HIV-1 Gag-Pol polyprotein provides a specific quantitative prediction for the cell surface presentation of the immunodominant KRWIILGLNK by HLA–B2705. By simulating the range of peptides derived from Gag-Pol, representing a range of off-rates, we observe that the enhancement by tapasin is independent of peptide supply, instead being wholly determined by the peptide off- and on-rates (Fig. 7 C).

To illustrate how the peptide optimization model may be used in more realistic scenarios, we simulated the presentation of HIV-derived peptides using our models for the HIV-associated allele HLA–B2705 (B2705), and HLA–B4402 (B4402) for comparison. Peptides between 8 and 10 amino acids in length were identified from the Gag-Pol polyprotein (UniProt; accession P03367) and assessed for their off-rates using the BIMAS prediction algorithm [28]. For B2705, the slowest off-rate identified was for the immunodominant KRWIILGLNK (positions 262–272) epitope [29] (off-rate: 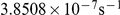). For B4402, the allele parameters of the BIMAS algorithm were not available, so we quantified off-rates based on the BIMAS algorithm parameters for the closely related allele B4403. The off-rates identified were generally higher than for B2705 (Fig. 7 A). When comparing simulations of B4402 in the presence and absence of tapasin, the highest affinity peptide AETGQETAY (positions 1250–1258; off-rate: 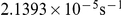) was enhanced by a factor of 445 by tapasin (Fig. 7 B), though cell surface presentation was over 25 times less than the presentation of KRWIILGLNK by B2705 (Fig. 7 B). Despite B2705 being a relatively tapasin-independent allele [6] (Fig. 4), tapasin significantly enhanced presentation of peptide KRWIILGLNK by a factor of 120 (Fig. 7 C).

## Discussion

The optimization of peptide-MHC class I complexes at the surface of antigen presenting cells is one of the key factors that determines the hierarchy of the T-cell response to a complex antigen [1]. Peptide optimization is also important for vaccine design, where vaccine peptides compete with endogenous peptides for presentation [1], [30]. In this paper we propose a dynamical systems model of MHC class I peptide optimization, which takes into account the supply of peptides in the cytosol, the affinity of peptides to MHC and the interactions between peptide and MHC at the different stages of the optimization process, both within the ER and at the cell surface. The model also incorporates the effects of tapasin, which is known to increase peptide optimization [5] and to affect different MHC class I alleles to different extents [6]. This variation in tapasin dependence may protect from viral immune evasion strategies such as tapasin inhibition by an adenovirus [31].

The dynamical systems model is firmly grounded in experimental data, and techniques already exist to measure many of the model parameters [5]–[8]. The model therefore allows a multitude of experimental results to be unified within a common framework, so that a range of mechanistic hypotheses can be formulated and tested. We derive a peptide filtering relation which, for the first time, provides a mechanistic explanation for experimental data on MHC class I peptide optimization, both over time [6] and at steady state [5]. Specifically, it suggests that tapasin enhances peptide off-rate in order to improve peptide optimization without significantly delaying the transit of MHC to the cell surface.

We have also shown that an allele-specific peptide on-rate is the most likely mechanistic explanation for differences in peptide optimization across HLA–B alleles. A possible interpretation is that differences in peptide on-rate are due to allelic differences in molecular conformation. For example, alleles such as B4402 could adopt a closed conformation, reducing the ability of peptides to bind MHC, while alleles such as B2705 could adopt a more open conformation, allowing peptides to readily bind MHC, as suggested in [32]. When tapasin binds to MHC the peptide binding groove may then adopt a peptide receptive conformation, allowing MHC to bind peptides more readily, as suggested in [20]. Although allelic differences in the conformation of MHC class I are largely peptide-independent, variations in the on-rates of different peptides have nevertheless been observed. These variations can be incorporated in future versions of the model by allowing a separate on-rate for each peptide. However, published estimates indicate that variations in the affinity of peptide-MHC interactions are mostly governed by variations in peptide off-rate [11], supporting our assumption that the on-rate is allele-specific and largely peptide-independent.

Although the current model makes a number of simplifying assumptions on the antigen presentation process, the model can be readily extended to incorporate additional details as more data are acquired. These details could include the explicit contribution of TAP transport, proteasomal cleavage and cytosolic protein abundance to ER peptide supply [26], [33], [34]. At present these mechanisms are only implicitly represented in the model via peptide-specific supply rates 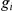. Further extensions could also include conformational changes in MHC [7], and chaperones such as ERp57 and calreticulin which are known to influence total cell-surface presentation [15], [17]. Since the mechanisms by which additional chaperones interact with MHC class I are only partially known, we can investigate a variety of hypotheses by using our Information Theoretic framework to assess allele-specific chaperone-dependency. In the future, coupling model analysis with additional experimental measurements will enable quantitative predictions of peptide optimization for a wide range of MHC class I genotypes. Having a robust model, known to make accurate predictions, will improve our ability to assess the efficacy of vaccines involving multiple peptides, and will provide a quantitative means to prioritize different vaccination strategies.

The current work is part of a broader research programme to use experimental data to build credible mathematical models of immunological processes, ranging from relatively simple examples to complex systems such as organ-specific autoimmunity. The resulting models can then be used to make specific and testable predictions that relate directly to immunological function. Subsequent iterations offer an opportunity to refine or develop the models from the simple to the complex, or from the static to the time-resolved, at the molecular, cellular or organ level.

## Methods

### Ordinary differential equation representation of the dynamical systems model

The chemical reaction representation of the dynamical systems model of Fig. 2 is as follows:



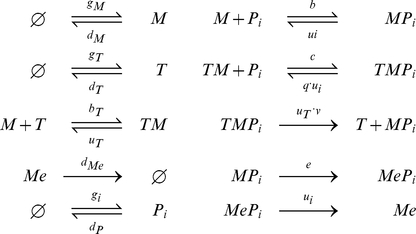



By assuming mass-action kinetics, we converted the system of reactions to a set of ordinary differential equations (ODEs), where 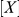 denotes the concentration of a given species 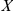 and 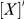 denotes rate of change in concentration.
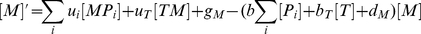
(3)

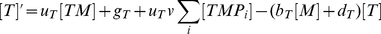
(4)

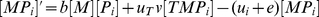
(5)

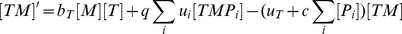
(6)

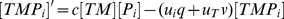
(7)

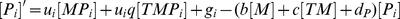
(8)

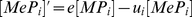
(9)

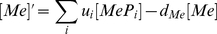
(10)


The dependent variables consist of MHC 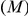, peptide 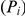, tapasin 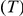, egressed MHC 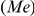 and the complexes formed between these elements. The equations denote a two-compartment system, comprising an ER model (3)–(8) and a cell surface model (9)–(10).

### Steady-state analysis of the ODE representation

Equation (1) was derived by considering the steady state (equilibrium) solutions of the ODE representation. By equating 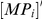, 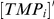 and 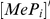 with zero, we obtained the following expressions for 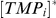 and 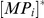:
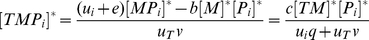
(11)

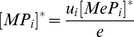
(12)


By substituting (12) in (11) we obtained the expression for the steady-state concentration of egressed MHC complexes with a given peptide 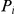 in equation (1)
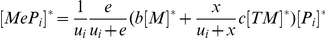



where 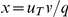. The equation incorporates the peptide filtering steps described in the main text, where 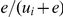 denotes the optimization of free MHC in the ER, while 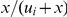 denotes the optimization of MHC bound to tapasin in the ER. For a given peptide with off-rate 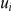, the optimization of free and tapasin-bound MHC is therefore fully determined by 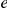 and 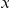, respectively. Moreover, MHC complexes optimized in the presence of tapasin will always be subject to an additional optimization step after tapasin unbinding. The equation also incorporates optimization of MHC at the cell surface given by 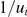, where peptides with a lower off-rate 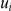 are more likely to remain bound to MHC.

### Optimizing model parameters with respect to experimental data

Heuristic search methods were used to fine-tune model parameters, based on the available experimental data. Our approach was to minimize a cost function, defined as the sum of the squared differences between experimental data 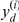 (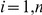) and corresponding model simulated output 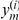, subject to an arbitrary proportionality constant 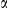. i.e.
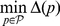
(13)

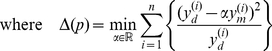
(14)


where 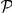 is the space of search parameters, which may be the full parameter set or a subset thereof. For the inner minimization problem (14), it is possible to assign an optimal 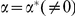, by equating the partial derivative of 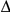 (with respect to 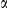) to 0.
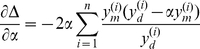
(15)

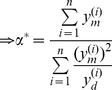
(16)


Approximate minimizers of the multi-dimensional objective function (13) were found using a Metropolis-Hastings (MH) algorithm.

During execution of the MH algorithm, a Markov chain of proposal parameter sets is formed. Starting from an initial parameter set 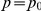 with associated objective function value 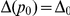, the algorithm iteratively searches neighboring parameter sets by accepting or rejecting new proposal parameter sets at each step. Neighboring points 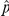 are proposed with probability 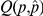, according to a *jump rule*

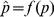
(17)


The chain moves to the new point 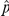 according to an acceptance criterion, which makes a probabilistic choice about whether to accept 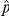. Given an observation 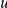 drawn from 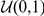, the proposal point is accepted providing
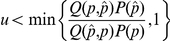



where 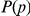 is the probability that the parameter set 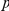 matches the data and 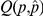 is the proposal density (which we fix to be symmetric). Assuming the deviations from the experimental data are Gaussian distributed and that 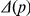 makes only small jumps, the acceptance ratio is approximately given by
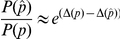



Note that when 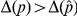 we always move to 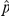, because the exponential function of a positive number is greater than one. The algorithm iterates until some termination condition is reached, such as a maximum number of iterations or a convergence condition. All parameter searches were performed using ‘Filzbach’, a software library for carrying out Metropolis-Hastings Markov chain Monte Carlo parameter estimation in C++ or C#. Filzbach is under development in the computational science lab at Microsoft Research Cambridge and is available for download, complete with a suite of example uses, via http://research.microsoft.com/science.

### Model selection using the Bayesian Information Criterion

To assess which model parameter(s) should be allele-specific, different hypotheses were compared using the Bayesian Information Criterion (BIC) [19]. The BIC is defined as
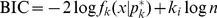
(18)


where 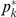 is the parameter set associated with model hypothesis 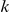 that maximizes the likelihood function 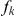, and 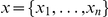 are the experimental observations. This is equivalent to minimizing the residual sum of squares, as in (14). It can be seen from this equation that the BIC penalizes the introduction of additional parameters.

### Solving the model equations

The model equations (3)–(10) were solved throughout using the CVODE routine [35], as part of SUNDIALS suite of numerical integrators [36]. During optimization with Filzbach, simulation code was written in C and compiled using Microsoft Visual Studio 2010. For plotting the simulations results, we used MATLAB and the SUNDIALS Toolbox for MATLAB. We have also provided an implementation of the model in SBML format for HLA–B (Protocol S1) and H2−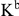 (Protocol S2).

### Computing the equilibrium concentrations

The equilibrium concentrations were computed from the model equations by equating the right hand sides to zero. This amounts to solving a system of nonlinear equations 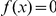, where 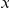 is the vector of concentrations and 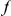 describes the fluxes resulting from production, degradation, binding, unbinding and ER egression. When using MATLAB, we used an implementation of the Levenberg-Marquhardt (L–M) search algorithm [37] to find solutions. When using the C implementation or in the case where the L–M algorithm did not converge to a non-negative solution, the ODEs were simulated using CVODE until the solution had not changed by more than 0.1% over a time interval of 1000 minutes. The latter method guarantees a non-negative solution providing the initial condition is also non-negative.

### Decay of MHC class I from the cell-surface with Brefeldin A

Brefeldin A (BFA) blocks anterograde traffic from the ER and thus the Golgi fuses with the ER. This prevents export of any newly synthesized class I from the ER to the cell-surface [38], [39]. BFA (Sigma, UK) was dissolved in methanol at 4 mg/ml for storage at 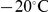 and used at 5 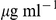. 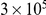 suspension cells were plated in 1 ml cell medium in a 24-well plate. 5 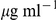 BFA was added for the indicated times and all the cells were harvested at the same time for flow cytometry. Cells were harvested for flow cytometry as previously described [5].

## Supporting Information

Protocol S1 An implementation of the model in SBML format for HLA-B.
(XML)

Protocol S2An implementation of the model in SBML format for H2-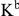.(XML)

Text S1 Supporting information**.** Here we outline in more detail the derivation of the model and the filter relation, and provide interpretation for parameter variation analyses. Included are 8 supplementary figures and 3 supplementary tables.
(PDF)
