# Supplementary material for: A Peptide Filtering Relation Quantifies MHC Class I Peptide Optimization
Source: PLoS Comput Biol. 2011 Oct 13;7(10):e1002144. doi: 10.1371/journal.pcbi.1002144 (PMC3195949; doi:10.1371/journal.pcbi.1002144)
Supplement: Text S1 — Supporting information. Here we outline in more detail the derivation of the model and the filter relation, and provide interpretation for parameter variation analyses. Included are 8 supplementary figures and 3 supplementary tables. (PDF) [file pcbi.1002144.s003.pdf]

Supporting Information: A peptide filtering relation quantifies MHC class I peptide optimization

Neil Dalchau[1,*], Andrew Phillips[1,*], Leonard D. Goldstein[2], Mark Howarth[3,4], Luca Cardelli[5], Stephen Emmott[1], Tim Elliott[3,6], and Joern M. Werner[6,7]

[1]Biological Computation Group, Microsoft Research, Cambridge, CB3 0FB, UK
[2]Department of Applied Mathematics and Theoretical Physics, University of Cambridge, Centre for Mathematical Sciences, Cambridge CB3 0WA, UK
[3]Faculty of Medicine, University of Southampton, Southampton SO16 6YD, UK
[4]Department of Biochemistry, Oxford University, South Parks Road, Oxford, OX1 3QU, UK
[5]Programming Principles and Tools Group, Microsoft Research, Cambridge, CB3 0FB, UK
[6]Institute for Life Sciences, University of Southampton, UK
[7]School of Biological Sciences, Faculty of Natural Sciences and the Environment, University of Southampton, Southampton, SO17 1BJ, UK
[*]Equal contribution

1 Stochastic model

A computational model of MHC class I peptide editing was developed using the Stochastic Pi Machine (SPiM) [1], a programming language for the modular description of concurrent stochastic systems. In this formalism, a system is specified as a collection of processes that communicate with each other through channels at stochastic rates. A protein in a defined state is identified as a process, and a channel is identified as a mechanism for altering this state. The properties of any system described in this language are simulated using an exact stochastic simulation algorithm [2]. Modularity of the approach allows the model to be extended with additional components, to a level of complexity that becomes analytically intractable. The three components of the peptide loading complex that are known to determine relative presentation were modeled explicitly (Fig. S1), namely peptides, MHC class I and tapasin. Briefly, peptides (Fig. S1A) are characterized by their ability to bind and unbind MHC with specific rates (Table S1). Only one peptide category is shown in Fig. S1, parameterized by the peptide off-rate i, though multiple peptide categories can be included, each with a different off-rate. The modularity of the approach allows an arbitrary number of peptide categories to be introduced without modifying the models for MHC or tapasin. The rates of peptide generation and degradation control the number of peptides in the system, implicitly approximating the behavior of the TAP transporter and the ER peptide degradation and efflux system. Tapasin is generated and degraded and has the ability to interact with MHC (Fig. S1B). MHC can interact both with peptides and with tapasin (Fig. S1C), where the effect of tapasin on peptide-MHC interactions is given by modulating the rates of peptide binding and unbinding (Table S1).

The SPiM language allowed for rapid exploration of alternative peptide editing models in a modular fashion. Once a suitable model was obtained, a corresponding set of chemical reactions was generated from the model according to the rules of the SPiM language [1] (see Methods).

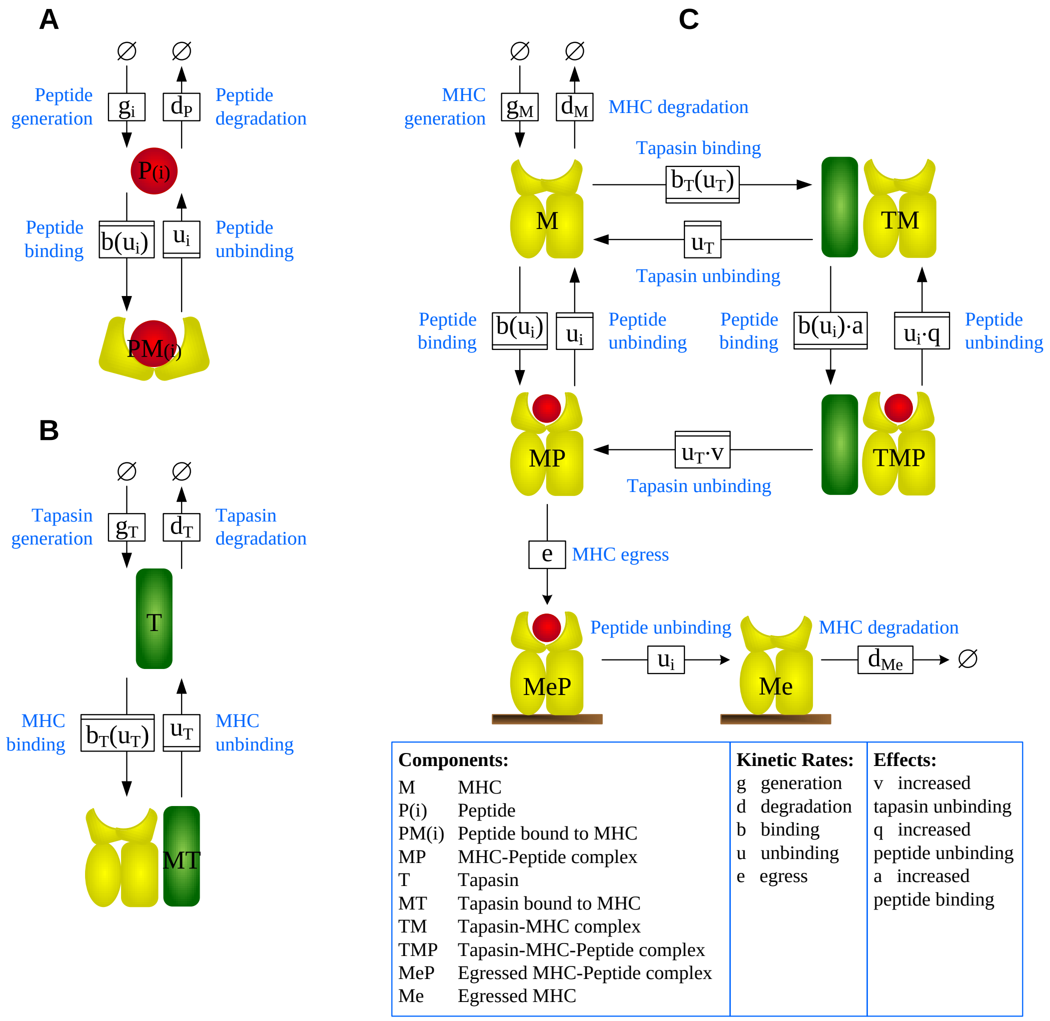

**Components:**		**Kinetic Rates:**	**Effects:**
M	MHC	g generation	v increased tapasin unbinding
P(i)	Peptide	d degradation	q increased peptide unbinding
PM(i)	Peptide bound to MHC	b binding	a increased peptide binding
MP	MHC-Peptide complex	u unbinding	
T	Tapasin	e egress	
MT	Tapasin bound to MHC		
TM	Tapasin-MHC complex		
TMP	Tapasin-MHC-Peptide complex		
MeP	Egressed MHC-Peptide complex		
Me	Egressed MHC		

Figure S1: A computational model of MHC class I peptide editing, programmed in SPiM.
Each of the graphs **A**, **B** and **C** describes the behavior of a molecular component in the system, and each shape represents a component in a particular state. Each box represents an action that a component can perform in order to change from one state to another. Actions such as production and degradation that affect only a single component are represented as square boxes. Interactions such as binding and unbinding that involve two components are represented as boxes with upper or lower lines, which denote complementary motifs. An overline denotes a send action, while an underline denotes a receive action, where send and receive are considered to be complementary. For example, a free peptide can send on b, while open MHC can receive on b, both in presence and absence of tapasin. The communication is parameterized by u_i, which represents the fact that MHC and peptide become bound after the interaction takes place, and can subsequently unbind by communicating on u_i. Each communication channel is associated with a rate, denoting the average number of reactions per unit time. **(A)** Peptides are actively transported into the ER at rate g_i, where they are degraded at rate d_P. A peptide binds to MHC by sending on b, and unbinds by receiving on u_i. **(B)** Tapasin is generated in the ER at rate g_T, where it is degraded at rate d_T. Tapasin binds to MHC by sending on b_T, and unbinds by receiving on u_T. **(C)** Free MHC is assembled in the ER at rate g_M, where it is degraded at rate d_M. Free MHC loads a peptide by receiving on b, and releases the peptide by sending on u_i. Peptide-loaded MHC leaves the ER at rate e, after which peptides can unbind at rate u_i. Peptides are assumed to immediately degrade following unbinding from MHC at the cell surface, which in turn is degraded at rate d_{Me}. Inside the ER, free MHC binds to tapasin by receiving on b_T, and unbinds by sending on u_T. MHC can also load a peptide when bound to tapasin, but can only egress after dissociating from tapasin. The degradation of MHC bound to tapasin and the degradation of loaded MHC in the ER are neglected. The potential effects of peptide and tapasin on MHC are modeled by allowing the reaction rates to be multiplied by constant factors.

2 ODE analysis of peptide editing

$$\frac{d[M]}{dt} = \sum_i u_i[MP_i] + u_T[TM] + g_M - (b\sum_i[P_i] + b_T[T] + d_M)[M] \quad \text{(S1)}$$

$$\frac{d[T]}{dt} = u_T[TM] + g_T + u_T v\sum_i[TMP_i] - (b_T[M] + d_T)[T] \quad \text{(S2)}$$

$$\frac{d[MP_i]}{dt} = b[M][P_i] + u_T v[TMP_i] - (u_i + e)[MP_i] \quad \text{(S3)}$$

$$\frac{d[TM]}{dt} = b_T[M][T] + q\sum_i u_i[TMP_i] - (u_T + c\sum_i[P_i])[TM] \quad \text{(S4)}$$

$$\frac{d[TMP_i]}{dt} = c[TM][P_i] - (u_i q + u_T v)[TMP_i] \quad \text{(S5)}$$

$$\frac{d[P_i]}{dt} = u_i[MP_i] + u_i q[TMP_i] + g_i - (b[M] + c[TM] + d_P)[P_i] \quad \text{(S6)}$$

$$\frac{d[MeP_i]}{dt} = e[MP_i] - u_i[MeP_i] \quad \text{(S7)}$$

$$\frac{d[Me]}{dt} = \sum_i u_i[MeP_i] - d_{Me}[Me] \quad \text{(S8)}$$

2.1 The proportion of peptides edited via the tapasin pathway

The peptide repertoire at the cell surface will generally be a mixture of peptides that were edited either in the presence or absence of tapasin. From equation 1 in the main text we observe that the ratio of egressed peptide-MHC complexes that are loaded in the presence and absence of tapasin is given by the ratio of $[TM]^*cx/(u_i + x)$ to $b[M]^*$. Since tapasin improves peptide editing, optimal editing will therefore be achieved in cases where $[TM]^*cx/(u_i + x) \gg b[M]^*$. This can be achieved if there is a large concentration of tapasin-bound MHC $[TM]^*$ compared with free MHC $[M]^*$. To assess partitioning between the two cohorts it would be desirable to express $[TM]^*$ in terms of $[M]^*$ directly, but the relation appears to be non-linear. Instead, we observe that since tapasin and peptide compete for free MHC, tapasin-assisted peptide editing will be favored by an increase in free tapasin. From conservation of components in (S1) – (S8) we observe that $[T]^* = g_T/d_T$. Thus, an increase in the concentration of tapasin-MHC complexes $[TM]^*$ can be obtained by increasing the ratio of the tapasin generation and degradation rates. Furthermore, increasing the rate b_T of tapasin binding to MHC or decreasing in the rate u_T of tapasin unbinding from MHC will also increase the ratio of $[TM]^*$ to $[M]^*$ so as to favor presentation via the tapasin pathway.

If we assume that tapasin binds strongly to MHC such that $[TM]^* \gg [M]^*$, the majority of peptides will be loaded via the tapasin pathway provided $cx/b(u_i + x)$ is not too low. The latter expression quantifies the extent to which MHC is able to load peptide in the presence of tapasin, which is known to be high in tapasin-dependent alleles [3]. Since tapasin tends to increase the rate at which peptides bind to MHC ($c \geq b$), it should be sufficient to ensure that $x = u_T \cdot v/q$ is not too low. Since peptides tend to increase the rate at which tapasin unbinds from MHC ($v \geq 1$), it should be sufficient to ensure that the rate of tapasin unbinding u_T is not too low and that the factor q is not too high. Otherwise, peptides will unbind before tapasin and will tend to favor editing via the non-tapasin pathway. Interestingly, if $[TM]^* \ll [M]^*$, for example due to rapid binding and unbinding of MHC and tapasin, in order for the majority of peptides to follow the tapasin pathway the rate of peptide binding in the presence of tapasin needs to be much greater than in the absence of tapasin ($c \gg b$).

2.2 The effect of high peptide turnover on peptide editing

According to equation 1 in the main text, the concentration of egressed peptide-MHC complexes $[MeP_i]^*$ is proportional to the steady-state concentration of peptides $[P_i]^*$. By considering the conservation of peptides we find that $[P_i]^* = (g_i - e[MP_i]^*)/d_p$. Since high affinity peptides are selectively egressed to the cell surface, the concentration of high-affinity peptides in the ER will be correspondingly reduced, effectively reducing the optimization of MHC. However, if we assume high peptide turnover characterized by high peptide generation and degradation rates [4], then the rate g_i of peptide generation in the ER will be much greater than the rate at which peptides are

transported from the ER, such that $g_i \gg e[MP_i]^*$. This implies that the concentration of peptides in the ER will depend almost exclusively on the peptide generation and degradation rates, with $[P_i]^* \approx g_i/d_P$. Intuitively, this means that any peptides that are egressed from the ER will be rapidly replaced, thereby reducing the effect of selective egress on the peptide repertoire and enabling maximum optimization.

2.3 The effect of tapasin on the peptide repertoire

If we assume that peptide loading takes place via the tapasin pathway and that peptides have a high turnover in the ER [5], we can simplify equation (1) as follows, where $C = c[TM]^*/d_P$:

$$[MeP_i]^* \approx \underset{}{C} \quad \underset{\text{Supply}}{g_i} \quad \underset{\text{Tapasin}}{\frac{x}{u_i + x}} \quad \underset{\text{ER}}{\frac{e}{u_i + e}} \quad \underset{\text{Surface}}{\frac{1}{u_i}} \tag{S9}$$

The equation corresponds to an upper bound on peptide optimization in the presence of tapasin. We can compute the corresponding equation in the absence of tapasin in a similar fashion, where the subscript KO denotes tapasin knock-out and $C_{KO} = b[M]^*_{KO}/d_P$:

$$[MeP_i]^*_{KO} \approx \underset{}{C_{KO}} \quad \underset{\text{Supply}}{g_i} \quad \underset{\text{ER}}{\frac{e}{u_i + e}} \quad \underset{\text{Surface}}{\frac{1}{u_i}} \tag{S10}$$

We use these equations to compute the proportion of a given peptide-MHC complex $[MeP_i]$ at the cell surface, both in the presence and absence of tapasin.

$$\frac{[MeP_i]^*}{\sum_k [MeP_k]^*} = \frac{g_i/(u_i(u_i+e)(u_i+x))}{\sum_k g_k/(u_k(u_k+e)(u_k+x))} \overset{e,x \to 0}{\longrightarrow} \frac{g_i/u_i^3}{\sum_k g_k/u_k^3}$$
$$\frac{[MeP_i]^*_{KO}}{\sum_k [MeP_k]^*_{KO}} = \frac{g_i/(u_i(u_i+e))}{\sum_k g_k/(u_k(u_k+e))} \overset{e,x \to 0}{\longrightarrow} \frac{g_i/u_i^2}{\sum_k g_k/u_k^2}$$

This defines a measure of peptide optimization, similar to the principles outlined in Fig. 3 of the main text, but also taking into account discrimination at the cell surface. In particular, discrimination will vary with $1/u_i^3$ in the presence of tapasin, and with $1/u_i^2$ in the absence of tapasin. Note that the proportion of egressed peptide-MHC complexes will also depend on the generation of available peptides, and that optimization is maximal as e and x tend to zero. We can use equations (S9) and (S10) to compute the effect of tapasin on the presentation of a given peptide P_i, given by the *fold enhancement* $F^{(i)}$:

$$F^{(i)} = \frac{[MeP_i]^*}{[MeP_i]^*_{KO}} = \frac{cx[TM]^*}{b(u_i + x)[M]^*_{KO}} \tag{S11}$$

From this we can compute the relative effect of tapasin on the presentation of a peptide P_i compared with a peptide P_j, given by the *relative fold enhancement* $F^{(i)}/F^{(j)}$:

$$\frac{F^{(i)}}{F^{(j)}} \approx \frac{1/(u_i + x)}{1/(u_j + x)} \overset{x \to 0}{\longrightarrow} \frac{1/u_i}{1/u_j} \tag{S12}$$

The equation shows that tapasin enhances the presentation of peptides according to the inverse of the peptide off-rate. This provides a mechanistic explanation for one of the key experimental findings [6], which showed that *tapasin enhances MHC class I peptide presentation according to peptide half-life*.

3 Time-dependent optimization

3.1 Model simulations for time-dependent optimization by MHC class I

Experiments were conducted [3] in which a fixed cohort of MHC molecules was labeled with a radioactive isotope, and the change in peptide cargo of the cohort was observed over time. These are generally referred to as *pulse-chase* experiments. To follow the thermostability profile of a fixed cohort of MHC class I complexes over time, cells were pulse-labeled with radioactive amino acids for 5 min and followed for 2 h, both in the presence and absence of

tapasin. We modeled the time-dependent optimization of a fixed cohort of MHC by replacing the molecule M in equations (S1) – (S8) with an indexed molecule M_k and by replacing the generation rate g_M with an indexed rate g_{M_k}, where $k \in \{1, 2\}$. We let M_1 and M_2 denote the unlabeled and labeled molecules, respectively. During the simulation, we switched from the generation of unlabeled molecules M_1 to the generation of labeled molecules M_2 for a short time period, to represent the radiolabelling step. By plotting the concentrations of all species where $k = 2$, we observed the progression of a fixed cohort of MHC molecules over time.

3.2 Deriving models for HLA-B4402, HLA-B2705 and HLA-B4405

Models were derived for the MHC alleles HLA-B4402, HLA-B2705 and HLA-B4405 so as to reproduce the dynamics observed in pulse-chase experiments [3]. In these experiments, samples of labeled MHC complexes were taken at various time points and the thermostability of complexes was measured by heating them to 4°C, 37°C and 50°C for 12 min. The amount of conformationally intact complexes that survived the heating process was used to indicate the proportion of complexes with low, medium or high affinity peptide. We therefore simulated three representative peptides P_{low}, P_{medium} and P_{high}. We assumed that only MHC complexes containing high affinity peptide were stable at 50°C, that complexes containing high and medium affinity peptides were stable at 37°C, and that all peptide-bound and empty complexes were stable at 4°C.

To identify the kinetic rates which were not measured, we applied our parameter estimation procedure to minimize the deviation of model simulations from the measurements of labeled MHC (Fig. 4 in the main text) and endoglycosidase-H (endoH) resistance (Fig. S4C). Consequently, we sought models (parameter sets) for each of the alleles B4402, B2705 and B4405. In order to understand how natural polymorphism in the MHC genetic region gives rise to variable time-dependent optimization, we allowed some parameter rates to vary between the different alleles (allele-specific) whilst keeping others fixed (allele-independent). This enabled us to evaluate a series of hypotheses which specify which reactions are allele-specific. We searched through the parameter space of each model for parameter sets which minimized the deviations between the available data for B4402, B2705 and B4405 and the corresponding simulated values (see Methods). As the number of allele-specific parameters varied between successive hypotheses, we used the Bayesian Information Criterion to assess the most representative model hypothesis for allelic variation. A Markov Chain Monte Carlo (MCMC) algorithm was used to sample from the parameter space, as implemented in the Filzbach software (see Methods).

3.3 Parameter variation analysis for time-dependent optimization

The peptide cargo of MHC undergoes time-dependent optimization, which is enhanced by tapasin [3]. Thus far, such optimization has only been measured at the whole cell level, and a distinction has not explicitly been made between optimization in the ER and optimization at the cell surface. To assess the potential impact of tapasin on time-dependent optimization, we used our model to explore the effects of the known biochemical interactions of tapasin (the tapasin factors c, q and v). We also investigated the effects of peptide-MHC egression (e) and tapasin unbinding (u_T) on time-dependent optimization.

The method we used was motivated by classical sensitivity analysis. Starting from the optimal parameter sets for the B4402, B2705 and B4405 MHC alleles, we changed each of the parameters c, q, v, e and u_T one at a time over a broad range. We defined the *degree of optimization* as the number of high affinity peptide-MHC complexes divided by total MHC (Fig. S5) and we computed the degree of optimization in the ER, at the cell surface, or combined ER and cell surface after 120 minutes. This allowed us to quantify how a particular factor influenced time-dependent optimization.

Parameter variation analysis indicated that optimization at the cell surface could only be improved by the considered parameters when reducing e for B2705 and B4405 (Fig. S5). Surprisingly poor optimization was observed prior to egression, and could be improved for all alleles by reducing q and e or increasing c, though only very slightly in the case of B4405 (Fig. S5; left panels). However, total combined optimization [3] could only be improved by reducing e, indicating that the cell surface values comprise the major contribution to the measured data. The cell surface dynamics associated with each allele nicely illustrate the hypothesized importance of the quantity $x = u_T v / q$, since in all three alleles it is possible to observe an inverse relationship between q and u_T or v. Although our analysis suggests that low x increases optimization, here we also observe that x should not be too small, suggesting a possible trade-off between improving steady state optimization and reducing the time taken to reach this optimization. In all cases, optimization was improved monotonically with c, the rate which determines the flux through the tapasin-dependent pathway.

Table S1: Parameter values for the M_b model.

Description	Parameter	Measured	Range	M_b
Production of tapasin in the ER	g_T		Fixed	1505
Degradation of tapasin in the ER	d_T		$10^{-6} - 10^{-2}$	1.726×10^{-3}
Production of MHC in the ER	g_M		Fixed	150.5
Degradation of MHC in the ER	d_M	$2 - 3 \times 10^{-4}$ s^{-1} [7, 8]	$10^{-6} - 10^{-1}$	7.989×10^{-5}
Degradation of MHC at the cell surface	d_{Me}	2.4×10^{-4} s^{-1} [6]	$10^{-6} - 10^{-1}$	9.329×10^{-5}
Degradation of peptides in the ER	d_P	0.13 s^{-1} [5]	Fixed	0.13
Binding of tapasin to MHC	b_T		$10^{-11} - 10^{-5}$	1.663×10^{-9}
Unbinding of tapasin from empty MHC	u_T		$10^{-6} - 10^{-1}$	1.185×10^{-6}
Binding of peptide to tapasin-bound MHC	c		$10^{-8} - 10^{-2}$	8.303×10^{-8}
Effect of tapasin on peptide-MHC unbinding	q		$1 - 10^{5}$	2.104×10^{4}
Effect of peptide on tapasin-MHC unbinding	v		$1 - 10^{3}$	936.3
Binding of peptide to empty MHC	b_{B4402}	$0.2 - 2 \times 10^{6}$ M^{-1}s^{-1} [7, 9]	$10^{-11} - 10^{-5}$	3.177×10^{-11}
	b_{B2705}		$10^{-11} - 10^{-5}$	1.945×10^{-9}
	b_{B4405}		$10^{-11} - 10^{-5}$	4.367×10^{-9}
Egression of loaded MHC from the ER	e		$10^{-4} - 1$	0.1142
Unbinding of peptides from MHC	u_{low}	$7.8 \times 10^{-6} - 4 \times 10^{-3}$ s^{-1} [9]	$10^{-8} - 10^{-2}$	8.764×10^{-4}
	u_{medium}		$10^{-8} - 10^{-2}$	5.658×10^{-6}
	u_{high}		$10^{-8} - 10^{-2}$	4.177×10^{-7}
Active transport of peptides into ER	g_{low}	$1.3 - 3.3 \times 10^{-6}$ M s^{-1} [5]	$1 - 10^{5}$	2.093×10^{4}
	g_{medium}		$1 - 10^{5}$	1.759×10^{4}
	g_{high}		$1 - 10^{5}$	1.064×10^{4}

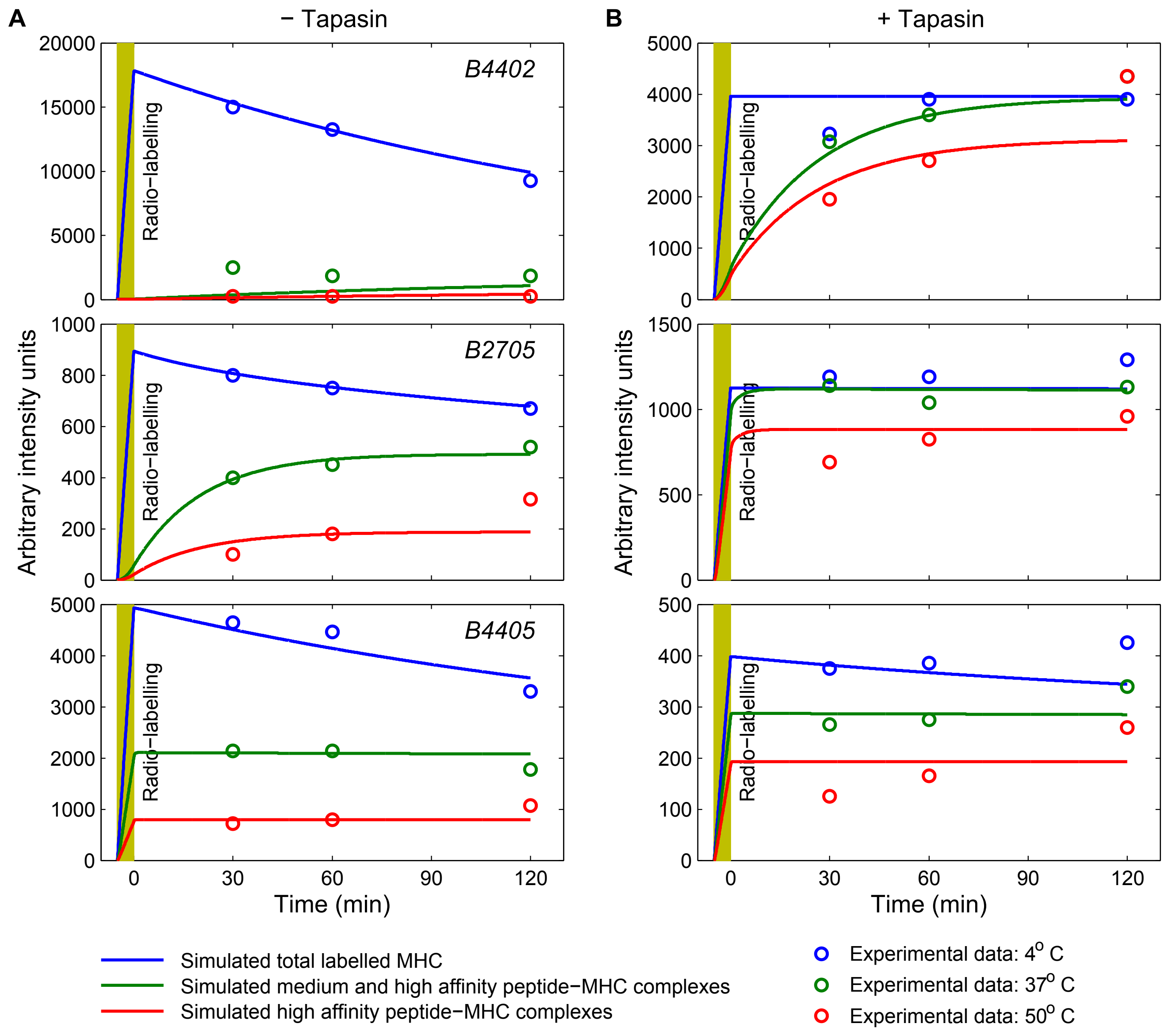

Figure S2: Simulation of the $M_{b,e}$ model hypothesis for MHC polymorphism.
A labeled cohort of MHC complexes was simulated using the model of Fig. 2 in the main text, by switching from generation of an unlabeled MHC population to a labeled population for the time interval indicated by the yellow blocks. The plots represent the sum of the concentrations of all peptide-MHC complexes at each time-point (blue), the sum of medium and high affinity peptide complexes (green) and just the high affinity peptide complexes (red). Corresponding experimental results [3] are also reported (circles). Simulations were conducted for representative low, medium and high affinity peptides with dissociation rates. The model was calibrated using different parameter sets for B4402, B2705 and B4405 alleles, both in the presence and absence of tapasin. The rate constants were identical across the different allele models, except for the binding of peptide to empty MHC (b) and the egression of peptide-MHC complexes from the ER (e) (see Table S1 for rate values).

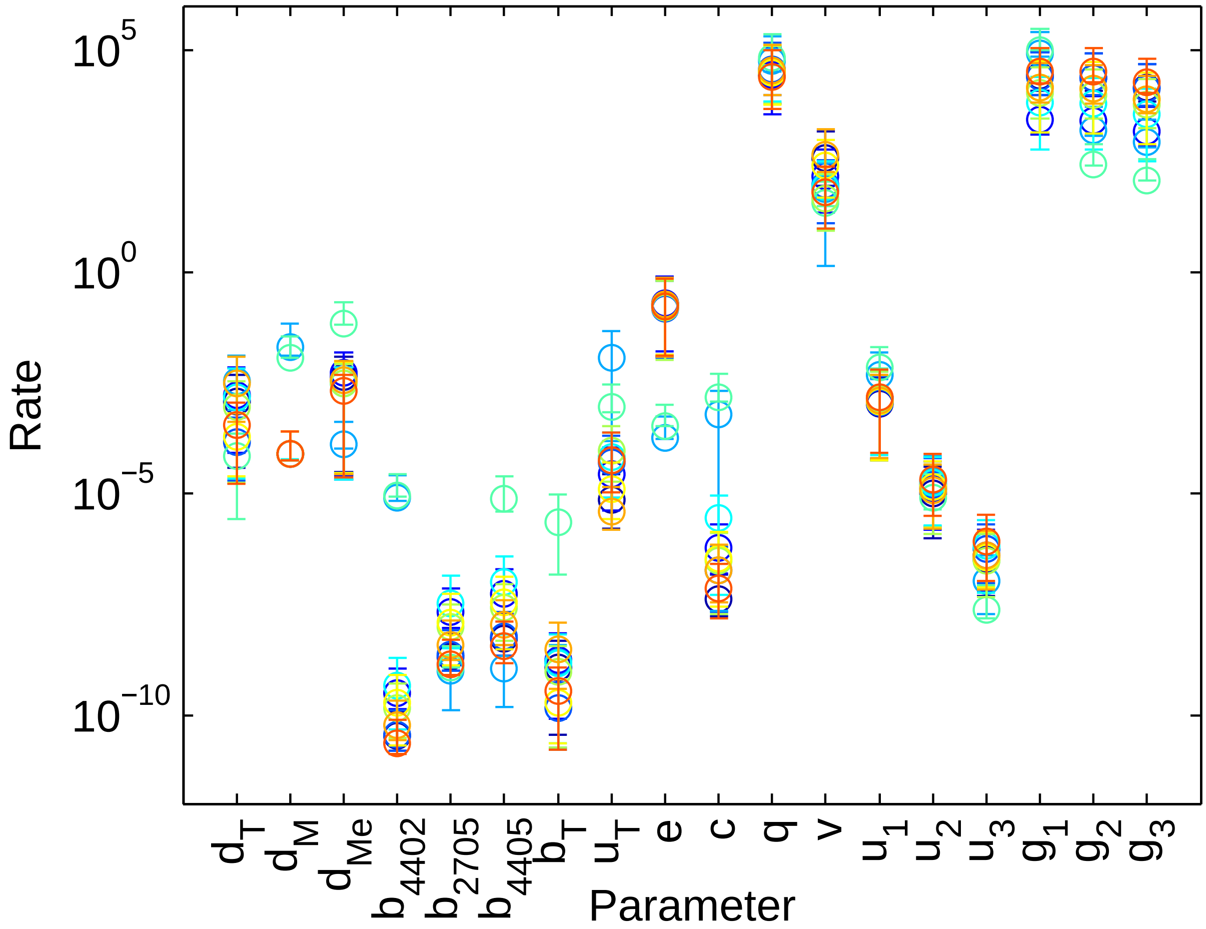

Figure S3: Posterior distribution convergence analysis for the M_b model.
The MCMC algorithm was instantiated 10 times independently (with random initial parameter vectors). Displayed here are the mean (circles) and standard deviations (error bars) of the posterior distributions for each parameter value, given the experimental observations. The posterior distributions were formed over 200’000 iterations, after a burn-in period of 200’000 iterations. Each color represents a different chain.

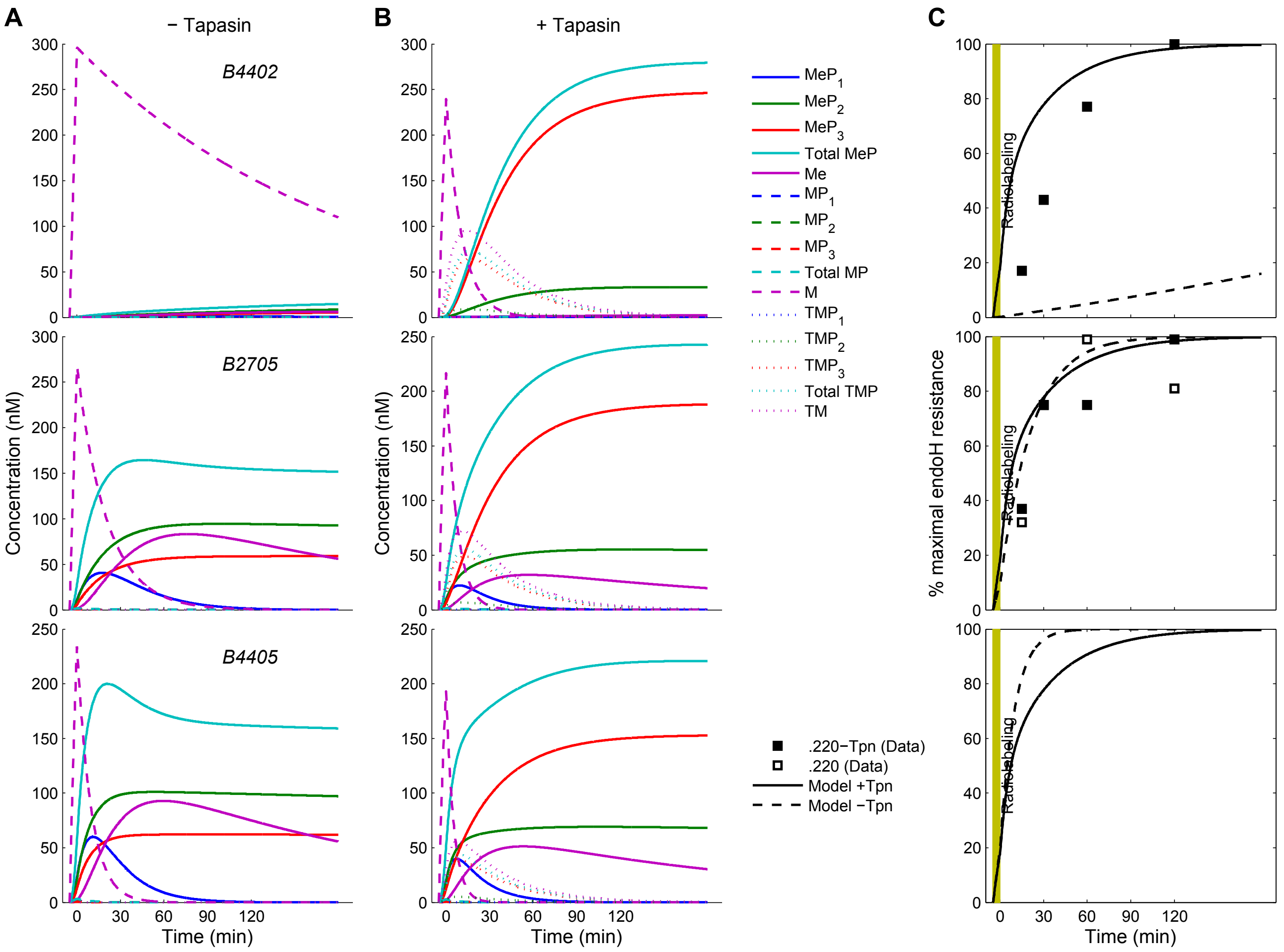

Figure S4: Detailed simulations of radiolabelling experiments [3].
Radio-labeling of MHC was simulated using two distinct populations of MHC molecules, an unlabelled population M_1 and a labeled population M_2. The molecule M in equations (S1) – (S8) was replaced with an indexed molecule M_k and the generation rate g_M was replaced with an indexed rate g_{M_k}, where $k \in \{1, 2\}$. The resulting equations were simulated by setting $g_{M_1} = g_M$ and $g_{M_2} = 0$ until the system approached steady state, and then setting $g_{M_1} = 0$ and $g_{M_2} = g_M$ for 5 minutes during the radiolabeling, before reverting back to the original values for g_{M_1} and g_{M_2} for the next 120 minutes. **(A, B)** Shown are labeled MHC and peptide-MHC complexes at the cell surface (Me), in the ER (M), and in the ER with tapasin bound (TM). Simulations conducted for absence **(A)** and presence **(B)** of tapasin. Peptides are indicated by P_i, where the subscript i represents low (1), medium (2) or high (3) affinity peptides. **(C)** Simulation of complex transit to the cell surface in the presence (solid lines) and absence (broken lines) of tapasin, corresponding to % maximal endoH resistance. Calculated as total cell surface MHC divided by maximum total MHC over the whole simulation. Corresponding measured data-points [3], comparing absence (open squares) with presence (closed squares) of tapasin.

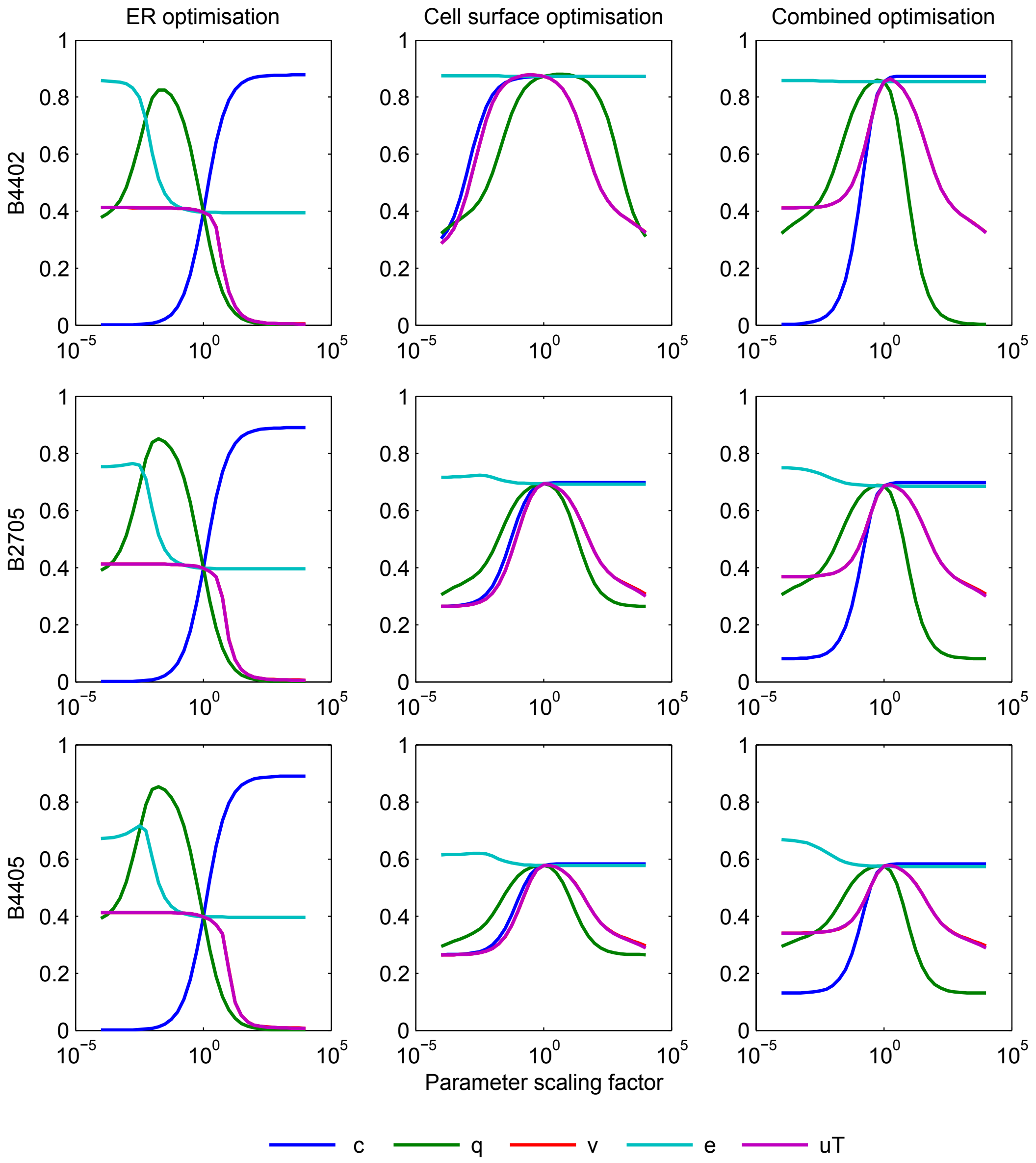

Figure S5: Parameter variation analysis for time-dependent optimization.
Radio-labeling was performed in silico by simulating two distinct MHC populations, as described in Fig. S4, assuming representative low, medium and high affinity peptides P_{low},P_{medium}, P_{high}, respectively. The degree of optimization was computed for the models of MHC alleles B4402 (top), B2705 (middle) and B4405 (bottom) 120 minutes after radio-labeling. Optimization was computed in the ER (left panels; $\frac{[MP_{\text{high}}]+[TMP_{\text{high}}]}{[M]+[TM]+\sum_i([MP_i]+[TMP_i])}$), at the cell surface (centre panels; $\frac{[MeP_{\text{high}}]}{[Me]+\sum_i(MeP_i)}$) and as a combination of the two (right panels; $\frac{[MP_{\text{high}}]+[TMP_{\text{high}}]+[MeP_{\text{high}}]}{[M]+[TM]+[Me]+\sum_i([MP_i]+[TMP_i]+[MeP_i])}$), for $i \in \{\text{low}, \text{medium}, \text{high}\}$. In each case, simulations were performed whereby parameters c, q, v, e and u_T were multiplied independently by a factor ranging between 10^{-5} and 10^5. Note that the results for varying v are hidden by near-identical results for varying u_T.

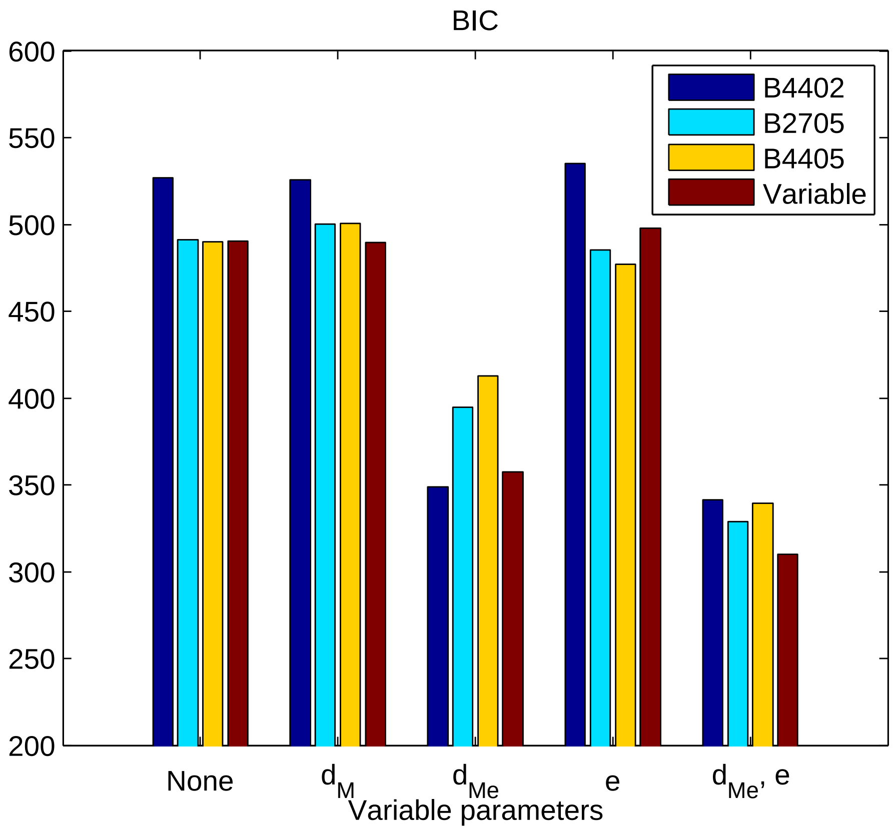

Figure S6: Identification of an MHC model for H2-K^b.
Filzbach (50'000 burn-in iterations followed by an additional 50'000 iterations for exploration) was used to find maximum likelihood estimate parameter sets from a range of base models. The bar groups distinguish the base model as having b fixed at either the values found for HLA-B4402, HLA-B2705 and HLA-B4405, or allowed to vary during the search. The parameter d_{Me} was fixed as $2.4 \times 10^{-4} s^{-1}$. Model classes where d_M, d_{Me}, e or both d_{Me} and e were allowed to vary were also investigated, starting from the B4402, B2705, or B4405 alleles, or with a variable b. The bars indicate the Bayesian Information Criterion (BIC) for the maximum likelihood estimates of each model.

4 Steady-state optimization

4.1 Competition between SIINFEKL variants and a population of endogenous peptides

To investigate how tapasin enhances MHC class I peptide presentation, we trained our HLA-B model to simulate the mouse MHC allele H2-K^b (K^b) using a combination of published [6] and novel experimental data. Steady-state presentation at the cell surface was measured [6] using mAb 25.D1 (D1), which specifically recognizes SIINFEKL peptide variants bound to K^b, and using mAb Y3, which recognizes all empty and peptide-occupied K^b. SIINFEKL is a high stability peptide derived from chicken ovalbumin, commonly used in the study of antigen presentation. We simulated the experiments [6] using three peptides. The first peptide was taken to be one of the SIINFEKL variants, so that the steady-state concentration $[MeP_i]^*$ corresponds with measurements of D1. Clearly, the number of presented complexes strongly depends on the pool of endogenous peptides with which it is competing, though this pool itself depends on the presence of tapasin. We chose to represent the pool of endogenous peptides by two peptides P_1, P_2, with dissociation rates $u_1 > u_2$, and supply rates g_1, g_2. In order to quantify suitable dissociation and supply rates for the endogenous peptides, we included - in our cost function for model identification - Y3 measurements following BFA treatment, which showed significant tapasin-dependence (see Fig. 6B in the main text). The cost function also comprised an EndoH term to calibrate the rate of egression ($\pm$tpn; see Fig. 6C in the main text), and terms for D1 and Y3 for calibrating SIINFEKL presentation (both $\pm$tpn; see Fig. 6D, E in the main text).

Based on the previously identified models for HLA-B, a suitable model for H2-K^b was identified using the

Table S2: Parameter values for the H2-K^b model.

Parameter	Value	Parameter	Value
b	2.755×10^{-10}	u_2	4.102×10^{-2}
d_{Me}	5.193×10^{-5}	g_1	1.831
e	7.071×10^{-4}	g_2	9.948×10^{6}
u_1	1.545×10^{-7}	g_0	24.325

Table S3: Levels of egressed peptide-MHC complexes measured by fluorescence of antibodies 25.D1 and Y3, in the presence and absence of tapasin. The fold enhancement with tapasin is also shown, where the FILKSINE fluorescence is subtracted from the 25.D1 fluorescence both in the presence and absence of tapasin. For each measured quantity, the experimental results are shown on the left, while the corresponding model simulation results are shown on the right. Experimental values are reproduced from [6].

	**D1 Antibody**				**Y3 Antibody**				**Fold**	
Peptide	.220-Tpn	Model	.220	Model	.220-Tpn	Model	.220	Model	.220	Model
SIINFEKL	1213	1205	31	60	1081	1197	1023	833	41.12	20.72
SIINFEKV	722	655	32	43	1032	1199	1019	833	23.62	15.59
SIINFEKM	604	414	72	34	1075	1199	1125	833	8.52	12.64
SIINYEKL	16	13	5	5	1031	1199	1113	833	3.53	2.98
FILKSINE	4		1.6		1120		1113		2.5	

Filzbach MCMC software (see Methods). H2-K^b is considered to be a moderately tapasin-dependent allele, which suggests it will be more dynamically equivalent to B4402 than B2705 and B4405. This hypothesis was initially tested by starting H2-K^b optimization from the parameter sets of B4402, B2705 and B4405, allowing only the parameters associated with the endogenous peptides (u_1, u_2, g_1 and g_2), the supply of SIINFEKL variants (g_0) to vary. As the degradation of empty MHC at the cell surface (d_{Me}) had been measured for H2-K^b, we fixed d_{Me} at the measured value (2.4×10^{-4}). The optimization procedure performed better for the B2705 and B4405 rates than for B4402 (Fig. S6), though comparing the simulations with the data indicated that none of the models were suitable. When enabling the allele parameter b to also vary, a minor improvement was observed over the HLA-B models with the smaller search space (Fig. S6). Next, we tried extending the search space to allow variation in parameters which could affect the system both in the presence and absence of tapasin (d_M, d_{Me} and e), one at time. A considerable improvement was observed when varying d_{Me}, with optimal BIC observed when fixing peptide binding at the B4402 rate (Fig. S6B). This might be explained by the simulation of the Y3 measurements after treatment with BFA (see Fig. 6B in the main text) strongly depending on the rates of peptide unbinding and surface MHC degradation. We therefore consider experimental data of this kind to be effective for determining d_{Me}.

The improvement gained from varying d_{Me} motivated us to also consider varying e, which we expected to be pivotal in reproducing the dynamics of complexes acquiring EndoH resistance (see Fig. 6E in the main text). The optimal $M_{b,d_{Me},e}$ model produced the best fit to the data in both the log-likelihood and BIC measures (Fig. S6A, B), so we retained this model as our candidate H2-K^b parameter set (see Table S2 for values). A comparison of measured and simulated D1 and Y3 measurements for SIINFEKL variants is presented in Table S3.

4.2 Peptide optimization is faster with enhanced dissociation than with delayed egress

Steady-state optimization was shown to depend critically on the factor x, which is the ratio between the rate of tapasin dissociation $u_T v$ and the rate enhancement q of peptide dissociation. Provided the approximation in equation (1) is valid, modifying $u_T v$ and q such that $u_T v/q$ remains constant should not alter the steady-state levels of cell surface peptide-MHC complexes, and thus the degree of peptide optimization. Therefore, considering steady-state measurements alone was not sufficient to identify $u_T v$ and q uniquely. We found that by also constraining the

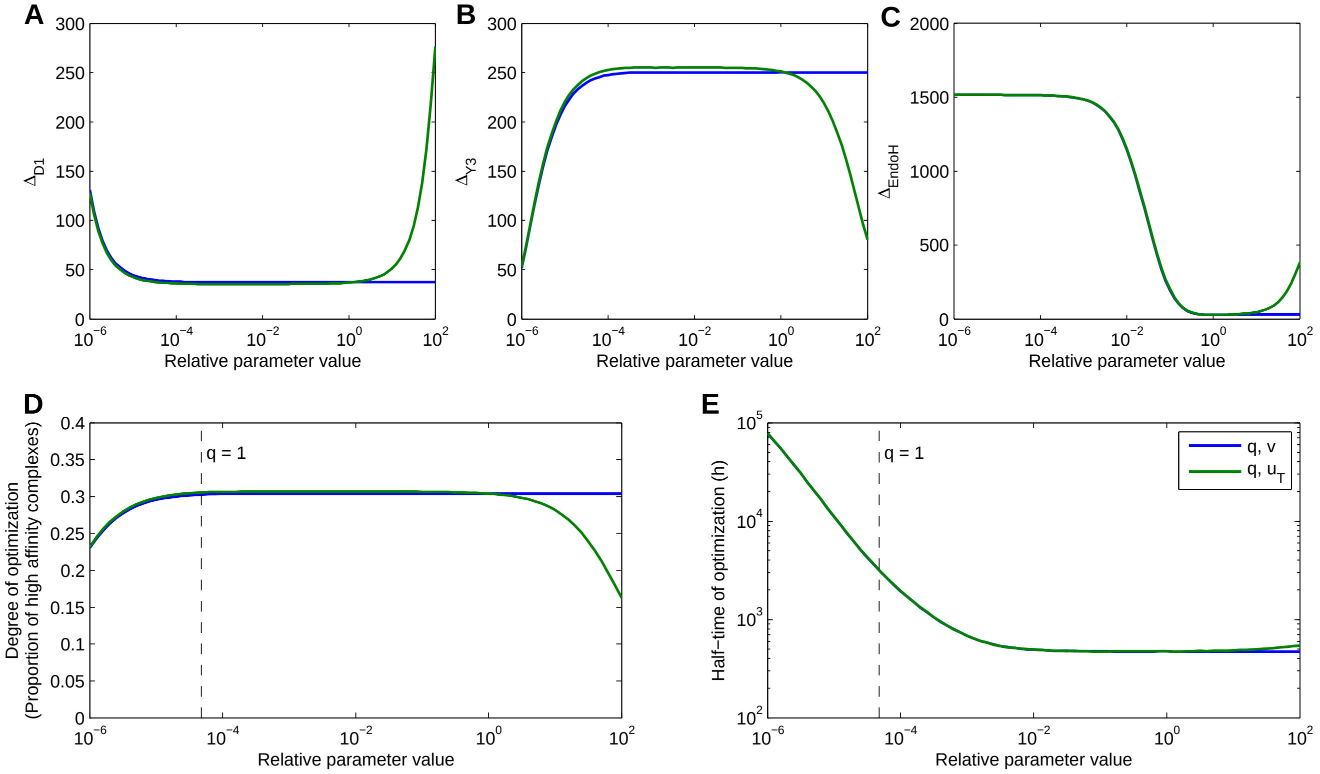

Figure S7: Decreasing q and $u_T v$ slows the rate of peptide optimization.
A parametric perturbation was applied to the model of Kb, which retains the approximate steady-state levels of cell surface peptide-MHC complexes. The parameters q and v (blue lines), or q and u_T (green lines) were multiplied by a constant factor (as indicated on the horizontal axis), which keeps $x = u_T v/q$ constant, and therefore the approximation in Eqn 1 fixed. The effect is shown on the cost function terms (**A**) Δ_{D1}, (**B**) Δ_{Y3} and (**C**) Δ_{EndoH}, (**D**) the degree of optimization (proportion of high affinity complexes at steady state), and (**E**) the half-time of the convergence to the steady-state. Also marked is the scale factor for which the scaled value of q is 1.

model parameters against data measuring endoglycosidase-H resistance (see Fig. 6E–G in the main text), which tracks the quantity of peptide-MHC complexes that have left the ER, we were able to identify optimal values for q and $u_T v$. We visualize this by plotting the cost function scores for D1, Y3 and EndoH against relative values of $u_T v$ and q, which illustrates the importance of the Δ_{EndoH} term in constraining the parameter set (Fig. S7A–C). Although a wide range of scale factors had no effect on the deviation from the D1 and Y3 data, decreasing q (and u_T or v) led to a large increase in the deviations from the EndoH data.

Through parameter estimation of the HLA-B alleles, we found that q was required to be high, with the rate of tapasin unbinding $u_T v$ being lower than the peptide off-rate $q u_i$ (Table S1). Therefore, tapasin achieves enhanced peptide optimization by increasing the peptide off-rate , rather than reducing the rate of tapasin unbinding. We hypothesized that by retaining complexes in the ER for longer through reduced tapasin unbinding, the time required to optimize the peptide cargo would be longer than if tapasin enhances dissociation. To test this hypothesis, we first calculated the degree of optimization at the cell surface over the range of parameter pairs (q, u_T) and (q, v) assessed for their impact on the cost function terms. The range of values for which the steady-state degree of optimization was invariant was identical to the range for which the steady-state cost function terms Δ_{D1} and Δ_{Y3} were invariant (Fig. S7A, B, D). In particular, decreases in the optimal parameter values down to a factor of approximately 10^{-4} were permissible, while only increases in (q, v) were permissible. We next examined the rate of optimization by computing the half-time of the approach to steady-state optimization, with reference to an initial state where there are no peptides, MHC or tapasin molecules. We found that decreases in the optimal parameter values down to a factor of approximately 10^{-2} did not change the half-time of optimization, though further decreases led to increasingly longer half-times, and therefore increasingly slower convergence to steady-state optimization (Fig. S7E). Therefore, there is an observable motivation for tapasin to effect optimization of the MHC class I peptide cargo through enhanced peptide-MHC dissociation, rather than retaining complexes in the

ER. This provides an explanation for the mechanism by which tapasin improves peptide optimization.

4.3 Parameter variation analysis

In order to understand how the model parameters relate to peptide optimization, we plotted the degree of optimization for the endogenous peptides P_1 and P_2 (Fig. S8A). Similar to the analysis in Section 3.3, each of the parameters c, q, v, u_T and e were varied up to five orders of magnitude above and below their candidate values for comparing the degree of optimization with the candidate model. We first found that total optimization (ER + cell surface) could be improved slightly by increasing c (Fig. S8A). In fact, optimization was an increasing function of c. In contrast, we found that the nominal values of u_T, v and q were very close to optimal in terms of peptide optimization. As both increases and decreases in these parameters lead to a decline in optimization, there must be trade-offs associated with these processes. For instance, increasing q enables more peptides to be scanned by MHC per unit time, though comes at the cost of decreased throughput via the tapasin pathway. If fewer peptides are loaded via the tapasin pathway, then the cell surface peptide-MHC complexes are less optimized, despite high q (or low u_T, v) improving the selection of high affinity peptides within the ER.

An interesting behavior was observed as e was decreased. The degree of optimization within the ER improved, as did optimization at the cell surface. However, although total optimization could be improved by decreasing e to approximately 10% of its nominal value, any further decrease led to an overall decline in optimization. This occurs because decreasing e increases the relative contribution of the ER to the total quantity of peptide-MHC complexes, and in the ER there are relatively few high affinity complexes (degree of optimization ~ 10^{-2}) compared with the cell surface (degree of optimization ~ 10^{-1}). A reduction in e also increased total cell surface presentation, indicating that the number of complexes at the cell surface was not the primary factor determining the rate e (Fig. S8B). As e was not identified at the optimal value in terms of both optimization and total presentation, an alternative selection pressure must be acting against slow egression of peptide-MHC complexes from the ER. A likely possibility is that slow egression increases the time taken between the arrival of an antigen and the activation of the T cell receptor, which decreases the effectiveness of the class I pathway. To investigate this possibility, we computed the rate of ER exit of peptide-MHC complexes ($e\sum_i [MP_i]^*$), and found that this quantity asymptotes to a maximum near to the estimated value of e (Fig. S8C).

References

[1] Phillips A, Cardelli L (2007) Efficient, correct simulation of biological processes in the stochastic pi-calculus. In: Computational Methods in Systems Biology. Springer, volume 4695 of *LNCS*, pp. 184–199.

[2] Gillespie DT (1977) Exact stochastic simulation of coupled chemical reactions. J Phys Chem 81: 2340–2361.

[3] Williams AP, Peh CA, Purcell AW, McCluskey J, Elliott T (2002) Optimization of the MHC class I peptide cargo is dependent on tapasin. Immunity 16: 509–520.

[4] Blanchard N, Shastri N (2008) Coping with loss of perfection in the MHC class I peptide repertoire. Curr Opin Immunol 20: 82–88.

[5] Yewdell JW, Reits E, Neefjes J (2003) Making sense of mass destruction: quantitating MHC class I antigen presentation. Nat Rev Immunol 3: 952–961.

[6] Howarth M, Williams A, Tolstrup AB, Elliott T (2004) Tapasin enhances MHC class I peptide presentation according to peptide half-life. Proc Natl Acad Sci U S A 101: 11737–11742.

[7] Schneeweiss C, Garstka M, Smith J, Hütt MT, Springer S (2009) The mechanism of action of tapasin in the peptide exchange on MHC class I molecules determined from kinetics simulation studies. Mol Immunol 46: 2054–2063.

[8] Kienast A, Preuss M, Winkler M, Dick TP (2007) Redox regulation of peptide receptivity of major histocompatibility complex class I molecules by ERp57 and tapasin. Nat Immunol 8: 864–872.

[9] Gakamsky DM, Davis DM, Strominger JL, Pecht I (2000) Assembly and dissociation of human leukocyte antigen (HLA)-A2 studied by real-time fluorescence resonance energy transfer. Biochemistry 39: 11163–11169.

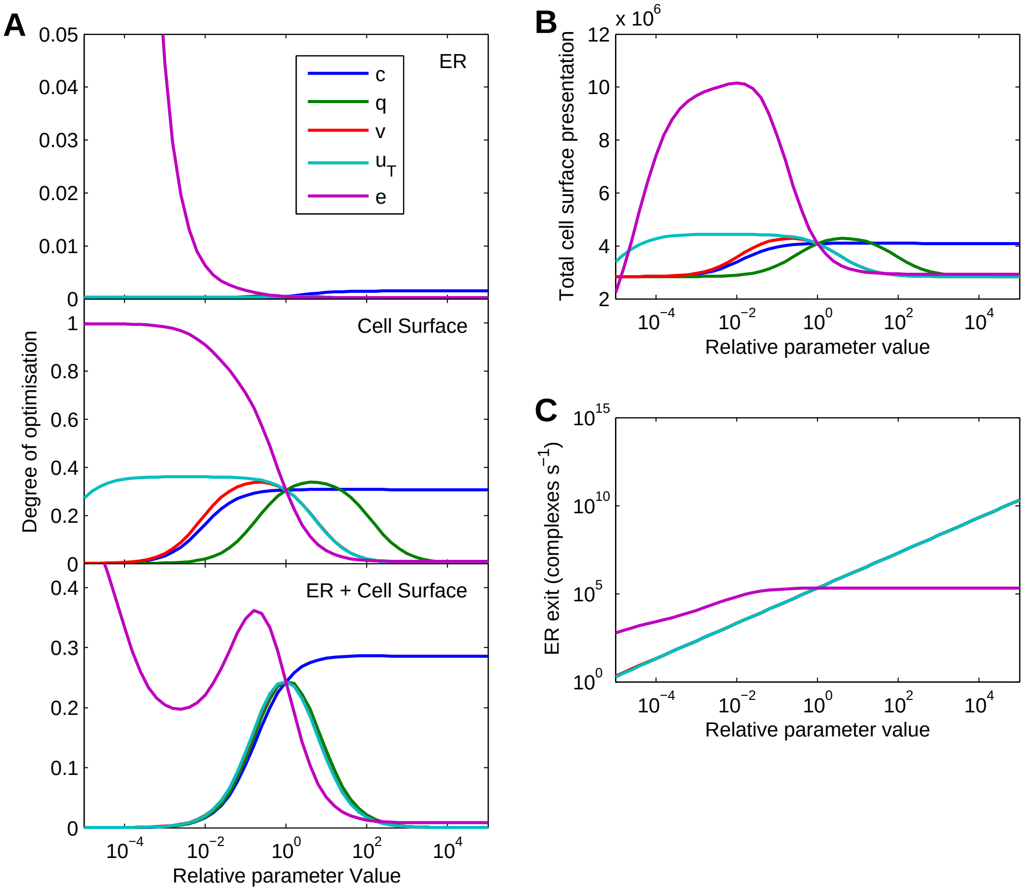

Figure S8: Parameter variation for the model of MHC allele H2-K^b.
Starting from the base values of the derived H2-K^b model (indicated by the open circles), each of the parameters c, q, v, u_T and e were increased and decreased up to 5 orders of magnitude to assess their impact on (**A**) the optimization of peptide cargo, (**B**) ER exit, and (**C**) total cell surface presentation. (**A**) The degree of optimization was computed as in Fig. S5, but for two peptides instead of three, using the low affinity endogenous peptide P_1 and the high affinity endogenous peptide P_2. Peptide dissociation and supply rates were as estimated. Optimization was considered within the ER (top panel), at the cell surface (middle panel) or as a combination of the two (bottom panel). (**B**) ER exit was computed as the total steady state number of peptide-MHC complexes multiplied by the rate of egression, i.e. $= e\sum_i [MP_i]^*$. (**C**) Cell surface presentation was computed as the total steady state egressed MHC, both peptide-bound and peptide-free, i.e. $= \sum_i [MeP_i]^* + [Me]^*$.
